# Lightweight Deep Learning Models with Explainable AI for Early Alzheimer’s Detection from Standard MRI Scans

**DOI:** 10.3390/diagnostics15212709

**Published:** 2025-10-26

**Authors:** Falah Sheikh, Ahmed Al Marouf, Jon George Rokne, Reda Alhajj

**Affiliations:** 1 Department of Computer Science, University of Calgary, Calgary, AB T2N 1N4, Canada; sheikhfalah.sheikhha@ucalgary.ca (F.S.); rokne@ucalgary.ca (J.G.R.); 2 Department of Computer Engineering, Istanbul Medipol University, Istanbul 34810, Turkey; 3 Department of Health Informatics, University of Southern Denmark, 5230 Odense, Denmark

**Keywords:** Alzheimer’s disease, deep learning, EfficientNetV2B0, MobileNetV2

## Abstract

**Background:** Dementia refers to a spectrum of clinical conditions characterized by impairments in memory, language, and cognitive function. Alzheimer’s Disease (AD) is the most common cause of dementia and it accounted for 60–70% of the estimated 57 million cases worldwide as of 2021. The exact pathology of this neurodegenerative condition is not fully understood. While it is currently incurable, progression to more critical stages can be slowed, and early diagnosis is crucial to alleviate and manage some of its symptoms. Contemporary diagnostic practices hinder early detection due to the high costs and inaccessibility of advanced neuroimaging tools and specialists, particularly for populations with resource-constrained clinical settings. **Methods:** This paper addresses this challenge by developing and evaluating computationally efficient lightweight deep learning models, MobileNetV2 and EfficientNetV2B0, for early AD detection from 2D slices sourced from standard structural magnetic resonance imaging (MRI). **Results:** For the challenging multi-class task of distinguishing between Cognitively Normal (CN), Early Mild Cognitive Impairment (EMCI), and Late Mild Cognitive Impairment (LMCI), our best model, EfficientNetV2B0, achieved 88.0% mean accuracy across a 5-fold stratified cross-validation (std = 1.0%). To enhance clinical interpretability and build trust, we integrated explainability methods, Grad-CAM++ and Guided Grad-CAM++, to visualize the anatomical basis for the models’ predictions. **Conclusions:** This work delivers an accessible and interpretable neuroimaging tool to support early AD diagnosis and extend expert-level capabilities to routine clinical practice.

## 1. Introduction

**Figure 1:**
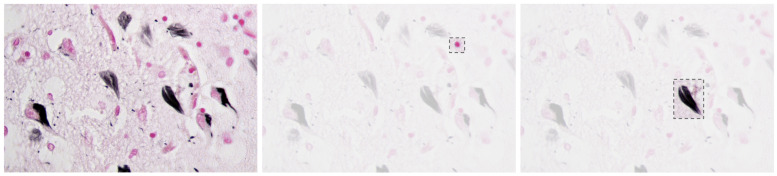
Alzheimer’s brain tissue with amyloid plaques (pink) and neurofibrillary tangles (black) [7].

Alzheimer’s Disease (AD) is a progressive neurodegenerative disorder that causes amnesia, aphasia, and cognitive decline [1], among other symptoms. The neuropathology of AD is marked by cerebral atrophy caused primarily by the accumulation of two abnormal proteins: beta-amyloid (Aβ) and tau [2,3]. Beta-amyloid aggregates extracellularly into plaques that disrupt neuronal communication, with the beta-amyloid 42 variant forming especially early and contributing significantly to the disease’s progression [1,4]. Concurrently, tau proteins missfold and accumulate intracellularly into neurofibrillar tangles, impairing axonal transport and contributing to synaptic dysfunction and neuronal death [2,5,6]. Figure 1 presents a histological section of brain tissue with Alzheimer’s markers, highlighting the characteristic amyloid plaques and neurofibrillar tangles, which are the hallmark pathological features of the disease.

Together, these pathological features degrade the neural networks of the brain and contribute to the clinical symptoms of the disease [8,9].

Alzheimer’s disease is the leading cause of dementia and it accounts for 60–70% of cases, affecting over 57 million people worldwide [10]. Although the disease is still incurable, early diagnosis allows for timely intervention that can significantly slow cognitive decline and improve quality of life [11]. However, current standard diagnostic methods, including Positron Emission Tomography (PET) scans, cerebrospinal fluid (CSF) analysis, and genetic testing, are costly and often only available at specialized centers, making them inaccessible in many resource-constrained clinical settings [12,13]. Table 1 below summarizes the primary neuroimaging and clinical assessment modalities.

Current Alzheimer’s disease treatment costs impose an economic burden that limits access to early diagnosis. In 2019, the global societal costs of dementia reached $1313.4 billion for 55.2 million people with dementia worldwide, corresponding to $23,796 per person affected [14]. Using alternative economic modeling approaches, the global macroeconomic burden of Alzheimer’s Disease and Other Dementias (ADODs) was projected to reach INT$14,513 billion from 2020 to 2050, equivalent to 0.421% of the annual global GDP [15]. Direct medical costs accounted for $213.2 billion (16%) of total costs, while direct social sector costs including long-term care represented USD 448.7 billion (34%) [14]. In the United States specifically, the total annual indirect cost of AD, including unpaid care and productivity losses, was estimated at USD 832 billion in 2024 [16]. The financial burden varies dramatically by disease severity. The total annual cost of AD per capita ranges from USD 468 in mild cases to USD 171,284 in severe cases [17], with indirect costs alone increasing from USD 36,934 for mild cognitive impairment to USD 145,250 for severe AD [16]. Advanced diagnostic procedures add substantial costs: chronic bi-weekly infusion treatments can cost USD 45,208 annually with additional caregiver burden of USD 6095, compared to oral treatments costing only USD 1983 with USD 457 in caregiver burden [18]. These escalating costs create significant barriers to early detection, particularly in resource-constrained settings where specialized neuroimaging and biomarker technologies remain inaccessible. Approximately 75% of people with AD will require nursing home care by age 80, compared to only 4% of the general population [19], highlighting the urgent need for cost-effective diagnostic tools that enable earlier intervention and potentially delay expensive institutional care.

While cognitive and clinical assessments, like MMSE and MoCA, are widely used as preliminary tools, they are notably prone to false positives [20]. For instance, one study found that the MMSE misclassified dementia in about 21% of cases, with errors strongly associated with lower education and visual impairment [21]. Furthermore, systematic reviews report that the MMSE suffers from ceiling effects in highly educated individuals and floor effects in those with low formal education, even after adjusting the score for educational level [20,22]. Moreover, psychiatric comorbidities such as depression, often referred to as “depressive pseudodementia”, can mimic AD symptoms and produce false positives when using limited screening tools [23,24].

**Figure 2:**
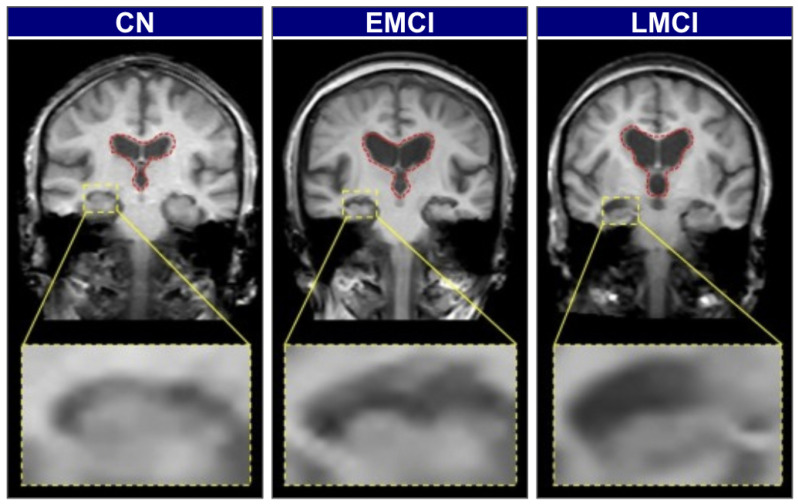
Representative coronal MRI slices illustrating the progression of neurodegeneration [25].

Biomarker-based methods offer greater diagnostic specificity but remain constrained by the same cost, invasiveness, and infrastructure limitations discussed above. To illustrate the visual and structural differences across stages of cognitive impairment, Figure 2 presents brain scans of individuals classified as cognitively normal (CN), early mild cognitive impairment (EMCI), and late mild cognitive impairment (LMCI). These stages reflect the gradual neurodegenerative progression associated with Alzheimer’s Disease.

In CN individuals, the brain exhibits a full volume with tight sulci, along with normal-sized ventricles [26,27]. As the condition progresses to EMCI, early structural alterations emerge, most prominently in the medial temporal lobes, where hippocampal atrophy is first observed. The hippocampus, a key component of the limbic system and part of the allocortex, shows this atrophy. This is often accompanied by a slight enlargement of the ventricles [27,28]. In the LMCI stage, these neurodegenerative changes become more pronounced. There is widespread cerebral atrophy, characterized by visibly widened sulci and significantly enlarged ventricles, reflecting substantial neuronal loss. The hippocampus, in particular, shows more advanced shrinkage, correlating with the more significant cognitive deficits observed at this stage [28,29]. These visually distinct and progressive patterns of atrophy on structural MRI provide an ideal quantitative target for automated classification models.

To address the diagnostic gaps left by these limitations, this paper introduces a practical and scalable solution: lightweight, explainable models that operate on standard structural MRI scans. We take advantage of computationally efficient deep learning architectures, specifically MobileNetV2 and EfficientNetV2B0, creating a tool that is both accurate and deployable on standard hospital hardware. Our methodology is grounded in data from the Alzheimer’s Disease Neuroimaging Initiative (ADNI), utilizing a cohort of 102 subjects with balanced representation across Cognitively Normal (CN), Early Mild Cognitive Impairment (EMCI), and Late Mild Cognitive Impairment (LMCI) categories. We implemented a rigorous preprocessing pipeline including skull stripping, RAS reorientation, and systematic extraction of 2D coronal slices, yielding a final dataset of 3000 image slices for training and evaluation.

To build clinical trust and provide transparent insights into model decision-making processes, we integrated a suite of explainability techniques, including Grad-CAM++ and Guided Grad-CAM++. These methods generate visual heatmaps that highlight the precise anatomical regions influencing a diagnosis, with our multi-scale attribution pipeline combining coarse-grained regional importance with fine-grained pixel-level attributions. Our best-performing model, EfficientNetV2B0 trained with augmentated data, achieved 92% overall accuracy and 91.8% balanced accuracy on the tri-class classification task. The explainability analysis confirmed that our models learn clinically meaningful representations, focusing on anatomically relevant regions including hippocampal and medial temporal structures that align with known AD pathophysiology. The main contribution of this work is, therefore, the development of an accessible, assistive tool for clinicians that extends expert-level diagnostic capabilities to routine practice, facilitating earlier and more reliable AD detection.

## 2. Related Works

Advancing AI-based diagnostics for Alzheimer’s disease demands an interdisciplinary research approach. This section reviews three key domains that inform our work. We begin by examining how deep learning has been applied to Alzheimer’s classification, particularly the shift from simple binary tasks to more complex multi-class problems that better reflect clinical reality. We then look at efficient neural network architectures, considering how researchers have tried to balance accuracy with computational constraints in medical imaging applications. Finally, we review explainable AI methods in healthcare, focusing on techniques that help clinicians trust model predictions. Through this review, we identify a gap in existing work and explain how our approach addresses the need for models that are simultaneously lightweight, accurate, and interpretable for early Alzheimer’s detection.

Deep learning has shown promise for analyzing neuroimaging data in AD diagnosis, though results vary significantly across studies. Early research focused primarily on binary classification tasks, such as distinguishing between patients with diagnosed AD and cognitively normal (CN) controls, where the best-performing models achieve accuracies up to 98.8% [30]. Such multiclass MRI classification approaches mirror Alzheimer’s disease progression, as demonstrated by recent AD-specific studies. For example, Olatunde et al. propose a framework for multiclass classification across prodromal AD stages using ensemble manifold regularization on structural MRI data [31]. For instance, Marcisz et al. [32] demonstrated that combining T1-weighted MRI radiomic features with MMSE scores improves multiclass discrimination among normal cognition, mild cognitive impairment, and Alzheimer’s disease. Similarly, Jasodanand et al. [33] developed a large-scale multimodal framework that fuses demographics, genetics, clinical measures, and multi-sequence MRI to predict amyloid/tau pathology, underscoring the advantage of integrative models in Alzheimer’s diagnostics.

Recent studies have demonstrated impressive results on these more challenging tasks. For instance, some research has explored sophisticated object detection frameworks. One investigation by (Wided & Abdelhamid in 2025) [34] using a YOLOv11 architecture reported 93.6% precision and 91.6% recall by strategically integrating structural MRI and Diffusion Tensor Imaging (DTI) data. Although models using genetic or clinical data alongside imaging often perform better, these additional data types are not always accessible in practice [35,36]. This makes it crucial to develop reliable models that work with standard sMRI scans alone [32]. A significant body of work has highlighted the challenges in this domain, particularly the need for large datasets and consistent preprocessing methods to ensure that models work reliably across different settings [37,38].

The deployment of deep learning models into real-world clinical workflows is often constrained by available computational resources [39]. This has stimulated the development of lightweight yet powerful neural network architectures that balance diagnostic accuracy with computational efficiency. However, the trade-off between efficiency and performance is not always linear [40]. Some comparative studies have found that in specific medical imaging contexts, heavier traditional architectures may still outperform their lightweight counterparts [41]. For example, research has shown that while lightweight models like MobileNet excel in natural image classification, their performance advantages may not consistently transfer to specialized medical imaging tasks, where domain-specific features and limited training data can favor deeper, more parameter-rich architectures [41]. This suggests that architectural choices must be carefully validated for each specific task and data modality. In contrast, ensemble methods that combine features from multiple architectures have shown great promise. A framework by Alruily et al. that integrated VGG16, MobileNet, and InceptionResNetV2 achieved 97.93% accuracy, 98.04% specificity, and 95.89% sensitivity, with a reported F1-score of 96.36% for the main results, showcasing the power of feature fusion [42].

A major barrier to clinical AI adoption is the “black box” problem [43]. Clinicians require transparent, verifiable decision-making processes to trust and act upon model predictions. Explainable AI (XAI) techniques address this need by revealing which image features or regions influenced classification decisions the most. In medical imaging, gradient-based attribution methods are widely used [44]. Grad-CAM uses gradients flowing into final convolutional layers to produce coarse-grained heatmaps highlighting broad regions of importance [45]. Grad-CAM++ improves localization through weighted combinations of positive partial derivatives, better handling multiple object instances [46]. For fine-grained explanations, Guided Grad-CAM++ combines coarse Grad-CAM++ maps with high-frequency detail from Guided Backpropagation, creating pixel-level, class-discriminative visualizations. Critical validation requires confirming these heatmaps align with known pathophysiology, such as hippocampal atrophy in AD [45,47].

Despite the significant progress made in each of the individual domains discussed above, AD classification, lightweight architectures, and XAI, there remains a notable gap at their intersection. While many studies have focused on achieving high accuracy using computationally intensive models, and a separate body of work has explored explainability, limited research has systematically integrated these three crucial elements [48,49]. Moreover, current clinical practice often fails to detect cognitive decline until later stages when intervention opportunities are limited [50,51,52]. Our investigation directly addresses these gaps by developing and evaluating computationally efficient, lightweight models for challenging, fine-grained early AD staging tasks (CN vs. EMCI vs. LMCI), coupled with rigorous anatomical validation using advanced explainable AI (XAI) techniques. This work delivers a framework that is simultaneously accurate, efficient, and interpretable, laying the groundwork for a clinically translatable tool that enables earlier recognition and intervention planning. Table 2 provides a comprehensive summary of the related works discussed in this section, comparing their datasets, architectures, tasks, explainability approaches, and key findings.

## 3. Materials and Methods

### 3.1. Dataset and Data Pre-Processing

Our methodology was designed to ensure reproducibility and clinical relevance, from data curation to model training and validation.

The study was based on data from the Alzheimer’s Disease Neuroimaging Initiative (ADNI) database [53]. To construct a well-defined cohort for the fine-grained tri-class classification of Cognitively Normal (CN), Early Mild Cognitive Impairment (EMCI) and Late Mild Cognitive Impairment (LMCI), we performed a targeted search during multiple phases of the ADNI project. The specific criteria used for data selection are detailed in Table 3. This precise process yielded a cohort of 102 subjects, balanced with 34 unique individuals per diagnostic class, with ages ranging from approximately 60 to 92 years, all with baseline T1-weighted sMRI scans acquired using the MPRAGE protocol [54,55].

These selection parameters were designed to ensure data quality and consistency across our tri-class classification task. We restricted our search to pre-processed images to maintain standardized acquisition protocols and reduce preprocessing variability that could confound model training. The inclusion of multiple ADNI project phases (ADNI, ADNI 1, ADNI GO, ADNI 2, ADNI 3, ADNI 4) was necessary to achieve sufficient sample sizes for each diagnostic category while maintaining temporal consistency in imaging protocols. We included baseline and early follow-up visits (ADNI-GO Month 3, ADNI-2 Month 6) to achieve sufficient sample sizes for balanced tri-class classification while maintaining diagnostic label consistency. Although this approach, combined with our slice-level data splitting, introduces potential subject-level leakage in our evaluation, it was necessary given the limited availability of prodromal AD cases in single-timepoint analyses. We explicitly acknowledge this methodological limitation in Section 6. The requirement for MPRAGE T1-weighted sequences ensured consistent image contrast and resolution, as this protocol provides optimal gray–white matter differentiation essential for detecting subtle structural changes in early cognitive impairment. Finally, we specified 3D acquisition to maintain full volumetric information during our subsequent 2D slice extraction process.

**Figure 3:**
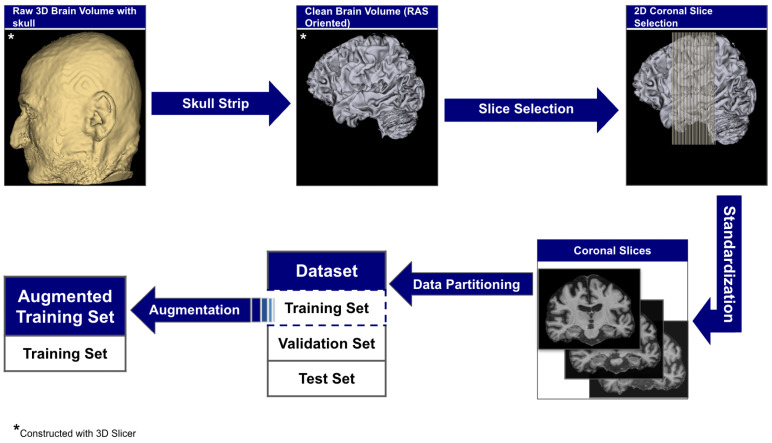
The end-to-end data preparation pipeline.

The raw 3D neuroimages underwent a rigorous, multi-stage preprocessing pipeline which is illustrated in Figure 3. To ensure our analysis focused purely on neuroanatomical structures, we first performed automated skull stripping using SynthStrip (FreeSurfer v8.0.0). This deep learning-based tool was specifically chosen because it operates without requiring spatial registration to a standardized brain template [56]. This registration-free approach is crucial as it preserves the native anatomical geometry of each subject’s brain and avoids the introduction of interpolation artifacts that can arise from spatial normalization [57]. Following skull stripping, each brain volume was reoriented to the RAS (Right–Anterior–Superior) canonical space to harmonize the data across the multi-site cohort.

From these aligned volumes, we extracted 2D slices exclusively from the coronal plane, providing a cross-sectional perspective along the ventral–dorsal axis. This view was deliberately selected as it offers the most direct and clinically relevant perspective for assessing the morphology of the hippocampus and surrounding medial temporal lobes, which are primary sites of early AD pathology [58,59]. This pipeline yielded a final dataset of 3000 2D image slices, 1000 per category. For each subject, we systematically extracted ~30 consecutive coronal slices centered around the volumetric midpoint of the coronal axis (slice range: $\left[i_{mid}-15,i_{mid}+15\right]$, where $i_{mid}$ represents the center index), specifically targeting the medial temporal region containing the hippocampus and surrounding structures critical for early AD pathology. We computed brain tissue bounding boxes using a fifth percentile intensity threshold, removed small objects (minimum size 256 pixels), applied binary closing (disk radius 5 pixels) to reduce noise, and excluded slices with zero intensity sums. This protocol extracted up to 30 slices per subject from the medial temporal region, yielding 1000 high-quality slices per diagnostic category across 102 subjects with consistent anatomical coverage. Slices were extracted as consecutive 2D arrays from the coronal plane without skipping frames, ensuring continuous anatomical coverage of hippocampal and periventricular structures throughout the medial temporal lobe region. To standardize the spatial dimensions for model input, we implemented an aspect ratio-preserving method where each slice was tightly cropped to the brain’s bounding box before being resized to fit within a 224 × 224 canvas, with zero-padding used to maintain anatomical proportions. Finally, each slice was intensity-scaled to the 0–255 range and stored in PNG format to simplify data handling and enhance accessibility.

Intensity normalization was applied on-the-fly during training using architecture-specific preprocessing functions from TensorFlow/Keras: MobileNetV2 scaled pixel values from [0, 255] to [−1, 1] via (x/127.5)−1, while EfficientNetV2B0 and DenseNet121 scaled to [0, 1] via x/255, matching their respective ImageNet pre-training protocols. No spatial registration to a common template (e.g., MNI152) or isotropic resampling was performed to preserve native anatomical geometry and avoid interpolation artifacts that could obscure subtle structural changes in early cognitive impairment; volumes maintained their original acquisition resolution (typical: 1.0 × 1.0 × 1.2 mm^3^). Since our method extracts 2D slices rather than performing 3D volumetric analysis, maintaining native through-plane resolution was prioritized to minimize preprocessing-induced artifacts. Quality control operated at both volume and slice levels: after skull stripping, volumes were visually inspected using orthogonal slice viewers and automatically flagged if brain tissue volume was <800 cm^3^ or residual skull signal exceeded 5% of total voxels above intensity threshold. At the slice level, we excluded slices with insufficient brain tissue coverage (<30% of image area) or extreme intensity distributions (mean intensity <10 or >200 after 8-bit scaling). Of 3060 extracted slices from 102 subjects (30 slices per subject), 60 slices (2.0%) were excluded due to insufficient tissue coverage (42 slices, 1.4%) or intensity artifacts (18 slices, 0.6%), yielding the final dataset of 3000 high-quality slices. No subjects were entirely excluded; quality control operated at the slice level to maximize data utilization while ensuring anatomical validity. This low failure rate (2.0%) compares favorably with reported preprocessing failure rates in similar neuroimaging studies (typically 3–7% [60]). The complete technical specifications for all preprocessing steps are detailed in Table 4.

This pipeline yielded a dataset of 3000 2D image slices (1000 per category) and was partitioned at the slice level into a training set of 1920 images (640 per class), a validation set of 480 images (160 per class), and a test set of 600 images (200 per class). The validation and test sets remained pristine and unchanged across all experiments to ensure a fair and consistent comparison of model performance.

The training set was used, however, in two different configurations to assess the impact of data augmentation, as detailed in Section 3.2.3. For one set of experiments, the 1920-image training set was subjected to on-the-fly transformations. These transformations included rotation (±10 degrees), shifts, shear, zoom (10%), horizontal flipping, and brightness adjustments (0.9–1.1), applied in real time during training. A detailed summary of the augmentation parameters is provided in Table 5.

### 3.2. Methodology

Our methodology was designed around two core principles: efficient computation for easy clinical use and clear interpretability for clinical trust. To show how the model is designed to align with the two principles, we describe our model architecture which has a multi-scale attribution pipeline for validation. We further specify the details of our training protocol.

#### 3.2.1. Model Architecture

We investigated and used two prominent lightweight convolutional neural networks, MobileNetV2 and EfficientNetV2B0, selected for their balance of high performance and low computational overhead. Both models were leveraged with transfer learning from their ImageNet pre-trained weights.

We froze the ImageNet pre-trained base networks and trained only the custom classification head (164K parameters) to mitigate overfitting risk given our limited dataset size of 3000 slices. With approximately 29–30 slices per subject across 102 subjects, full fine-tuning of millions of base parameters would likely overfit to subject-specific features rather than learning generalizable AD pathology patterns. The frozen feature extractors provide robust low-level and mid-level representations (edges, textures, shapes) learned from ImageNet’s diverse natural images, while the trainable classification head adapts these features to our specific tri-class AD classification task. This approach balances leveraging pre-trained knowledge with preventing overfitting on our relatively small medical imaging dataset.

MobileNetV2 achieves its efficiency by replacing standard convolutions with depthwise separable convolutions. This technique dramatically reduces computational cost by splitting a single complex operation into two simpler steps: one that filters features spatially and another that combines them across channels. This structure allows the network to learn rich features at a fraction of the computational cost when using traditional designs.

EfficientNetV2B0 is based on the principle of compound scaling, which simultaneously scales depth, width, and input resolution using a single, unified coefficient [61]. Rather than increasing just one of these dimensions, this balanced and coordinated approach leads to a more efficient and accurate architecture. The result is a network that delivers higher accuracy while maintaining a fixed computational budget, making it ideal for resource-constrained applications.

For the tri-class classification task, the terminal layers of each base network were replaced with a custom head composed of a Global Average Pooling (GAP) layer, a 128-unit Dense layer with ReLU activation, and a final three-unit Softmax layer to output class probabilities.

**Figure 4:**
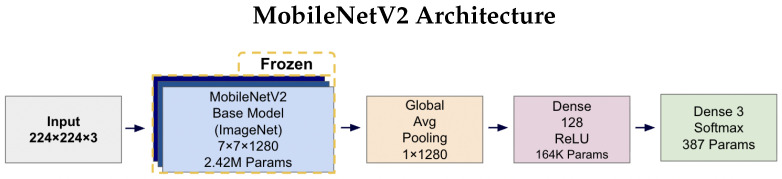
MobileNetV2 architecture diagram showing the frozen ImageNet pre-trained base model (2.42 M parameters) with a custom trainable classification head. The input 224 × 224 × 3 images pass through the frozen MobileNetV2 base, producing 7 × 7 × 1280 feature maps. These are processed by Global Average Pooling to create a 1 × 1280 feature vector, followed by a 128-unit Dense layer with ReLU activation (164K parameters) and a final three-unit Softmax layer (387 parameters) for tri-class prediction.

**Figure 5:**
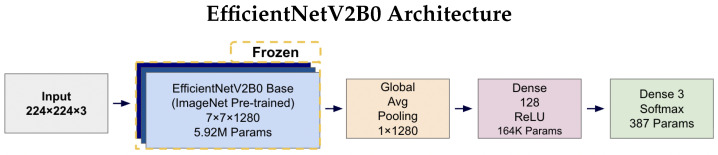
EfficientNetV2B0 architecture diagram illustrating the frozen ImageNet pre-trained base model (5.92 M parameters) with identical custom classification head as MobileNetV2. The compound scaling approach in EfficientNetV2B0 results in a higher parameter count in the base model while maintaining the same output dimensions (7 × 7 × 1280) and identical downstream processing through Global Average Pooling, 128-unit Dense layer, and 3-unit Softmax output for tri-class classification.

Figure 4 and Figure 5 illustrates the complete architectures for both models. The key architectural difference lies in the base models: MobileNetV2 employs depthwise separable convolutions for computational efficiency (2.42 M parameters), while EfficientNetV2B0 uses compound scaling techniques for enhanced accuracy (5.92 M parameters). Both architectures share identical custom classification heads, ensuring consistent output processing and enabling fair comparison of the base model performance. The frozen base models leverage ImageNet pre-trained weights, while only the classification head parameters (164K + 387 = 164.4K total) are trained on the AD classification task.

#### 3.2.2. Anatomical Validation with Explainability

A correct prediction is insufficient as we must validate that the model’s internal representations are clinically meaningful. Therefore, we developed a multi-scale attribution pipeline designed to produce robust and anatomically grounded explanations by synthesizing information from two complementary views.

The first stage addresses coarse anatomical localization. We employed Grad-CAM++ to generate a class-discriminative heatmap from the final convolutional layer. This provides a low-frequency view, answering the high-level question: “Which broad anatomical regions did the model consider relevant for its prediction?”

The second stage targets fine-grained feature attribution. Here, we used Guided Grad-CAM++, which combines the coarse localization of Grad-CAM++ with the high-frequency gradient information from Guided Backpropagation. By intersecting these two signals, the method highlights fine-grained features within the broader relevant regions, addressing the more specific question, “Within those regions, which pixels or textural features most influenced the model’s decision?”

To produce a robust visualization that reduces the limitations of individual methods, the normalized attribution maps from both scales were averaged to create a Consensus Map. This map highlights features that are consistently important at both the coarse anatomical level (Grad-CAM++) and the fine-grained pixel level (Guided Grad-CAM++). Finally, a brain mask was applied to all visualizations to eliminate background noise and focus interpretation on the relevant neuroanatomy.

#### 3.2.3. Training Details

The models were trained to minimize the Categorical Cross-Entropy loss function. We utilized the Adam optimizer with an initial learning rate of $10^{-3}$ and default beta parameters. Training was conducted on an NVIDIA A100 GPU. To ensure we selected the most generalizable model and prevented overfitting, we implemented an early stopping protocol. This procedure monitored the validation loss and terminated training after seven consecutive epochs of no improvement, after which the model weights from the best-performing epoch were restored for final evaluation. Complete hyperparameter specifications are detailed in Table 6.

#### 3.2.4. Inference Performance Characteristics

To validate clinical deployment feasibility, we benchmarked inference performance on standard hardware. Table 7 presents comprehensive metrics for all model configurations on both CPU and GPU environments.

MobileNetV2 demonstrated the fastest inference time at 174.6 ms per image on CPU, enabling the processing of 687 studies per hour, while EfficientNetV2B0 achieved 345.5 ms per image with a throughput of 347 studies per hour. Memory footprint remained modest across all architectures, with peak RAM usage of 3.3–5.5 GB and GPU VRAM utilization of 2.5–4.5 GB, well within the constraints of standard clinical workstations. These metrics confirm that both lightweight architectures meet the latency and resource requirements for real-time clinical deployment.

To ensure robust performance evaluation and address potential bias from single train–test splits, we employed stratified 5-fold cross-validation as our primary evaluation methodology. The complete dataset of 3000 slices was partitioned into 5 stratified folds, maintaining class balance across all partitions. For each fold, models were trained on 2400 samples (800 per class) and evaluated on 600 samples (200 per class). This yielded 5 independent performance estimates per configuration, enabling calculation of mean metrics with standard deviations to quantify result stability.

To evaluate the impact data augmentation has on model performance, we trained each architecture, MobileNetV2 and EfficientNetV2B0, on two data configurations: (1) the original dataset consisting of the base 3000 coronal slices (1000 per class) without oversampling or augmentation, and (2) an augmented dataset where on-the-fly data augmentation techniques described in Section 3.1 and detailed in Table 5 were applied directly to the 1920-image training set during training without any class resampling or oversampling.

In both configurations, the models were evaluated against the same pristine validation (480 images) and test (600 images) sets. This experimental design yielded four total models for a comprehensive comparative analysis. The code used for the research can be found in https://github.com/falahsheikh/eAlz (accessed on 22 October 2025).

All reported performance metrics including accuracy, sensitivity, specificity, PPV, NPV, AUC, and Brier scores were computed at the slice level using the pristine 600-slice test set (200 per class), consistent with our 2D classification framework. One-vs.-rest (OvR) AUC values were calculated for each class by treating it as the positive class against all others combined, with macro-averaging (unweighted mean across classes) and micro-averaging (weighted by class support) computed to assess overall discriminative performance. Calibration was evaluated using class-specific Brier scores, with lower values indicating better-calibrated probability estimates. Slice-level evaluation maximizes statistical power for rigorous architecture comparison and is standard practice in medical imaging studies with limited subject cohorts [30,34]. While subject-level aggregation through majority voting or probabilistic fusion would provide additional clinical validation, slice-level metrics enable precise quantification of architectural performance differences and augmentation effects.

## 4. Results

We systematically evaluated both architectures across original and augmented training configurations in the test set. All models were trained using categorical cross-entropy loss with Adam optimizer (learning rate $10^{-3}$) and early stopping (patience = seven epochs) [62,63].

### 4.1. Performance Overview

Table 8 presents comprehensive per-class metrics for all model configurations.

All configurations achieved strong performance (0.86–0.88 accuracy), with EfficientNetV2B0 augmented achieving the highest at 0.88.

Extending beyond classification accuracy, we conducted a comprehensive evaluation of discrimination and calibration performance across all model configurations. EfficientNetV2B0 (augmented) demonstrated better discriminative ability with macro-average AUC of 0.973 [95% CI: 0.963–0.982] and micro-average AUC of 0.973, compared to MobileNetV2 augmented (macro AUC: 0.970 [0.961–0.979], micro AUC: 0.970) and DenseNet121 augmented (macro AUC: 0.947 [0.933–0.960], micro AUC: 0.942). The original (non-augmented) configurations of our lightweight architectures achieved macro AUCs of 0.971 [0.961–0.980] for EfficientNetV2B0 and 0.962 [0.951–0.972] for MobileNetV2, demonstrating that data augmentation provided modest but consistent improvements in discriminative performance. Comprehensive discrimination and calibration metrics are presented in Table 9.

Calibration analysis with Brier scores revealed that EfficientNetV2B0 augmented achieved optimal probability calibration (Brier = 0.0588), followed by MobileNetV2 augmented (0.0628) and DenseNet121 augmented (0.0891). The original configurations showed slightly higher Brier scores (EfficientNetV2B0: 0.0643, MobileNetV2: 0.0706), confirming that augmentation benefits both discrimination and calibration.

Class-wise sensitivity, specificity, positive predictive value (PPV), and negative predictive value (NPV) metrics are presented in Table 10. The class-wise performance metrics reveal superior and balanced performance for EfficientNetV2B0 (augmented) across all diagnostic categories.

The class-wise performance metrics reveal superior and balanced performance for EfficientNetV2B0 (augmented) across all diagnostic categories. The model maintains excellent specificity across all classes (CN: 0.970, EMCI: 0.905, LMCI: 0.945), minimizing false positive diagnoses. MobileNetV2 (augmented) demonstrates similarly balanced performance with consistent sensitivity across categories (0.855–0.875) and uniformly high specificity (≥0.925). In contrast, DenseNet121 exhibits significant performance imbalance. Although achieving high LMCI sensitivity (0.900), it shows significantly lower sensitivity for CN and EMCI (both 0.775) and markedly reduced LMCI specificity (0.840) compared to the lightweight architectures. This pattern indicates DenseNet121 exhibits bias toward classifying cases as late-stage impairment, resulting in poor PPV for LMCI (0.738) despite reasonable NPV (0.944). Within our experimental framework, EfficientNetV2B0 demonstrated the most balanced performance across evaluation metrics, though validation on larger, more diverse cohorts is necessary to establish generalizability.

### 4.2. Training Dynamics

All configurations demonstrated stable convergence with early stopping triggered between 27 and 68 epochs. MobileNetV2 converged more rapidly (27–49 epochs) while EfficientNetV2B0 required extended training (30–68 epochs) to reach optimal performance. Augmented models showed smoother validation curves with reduced overfitting across both architectures. Detailed convergence metrics for all model configurations are presented in Table 11.

### 4.3. Architecture-Specific Analysis

In this section, we have presented the deep learning architecture-specific analysis based on the performance metrics. The MobileNetV2 and EfficientNetV2B0 performance, computational efficiency has been summarized.

#### 4.3.1. MobileNetV2 Results

**Figure 6:**
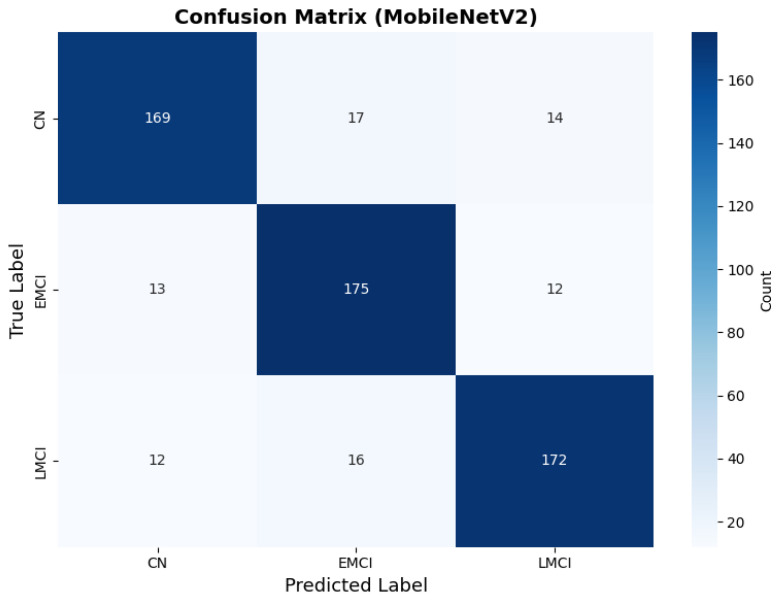
Confusion matrix showing strong diagonal classification for MobileNetV2 original configuration.

**Figure 7:**
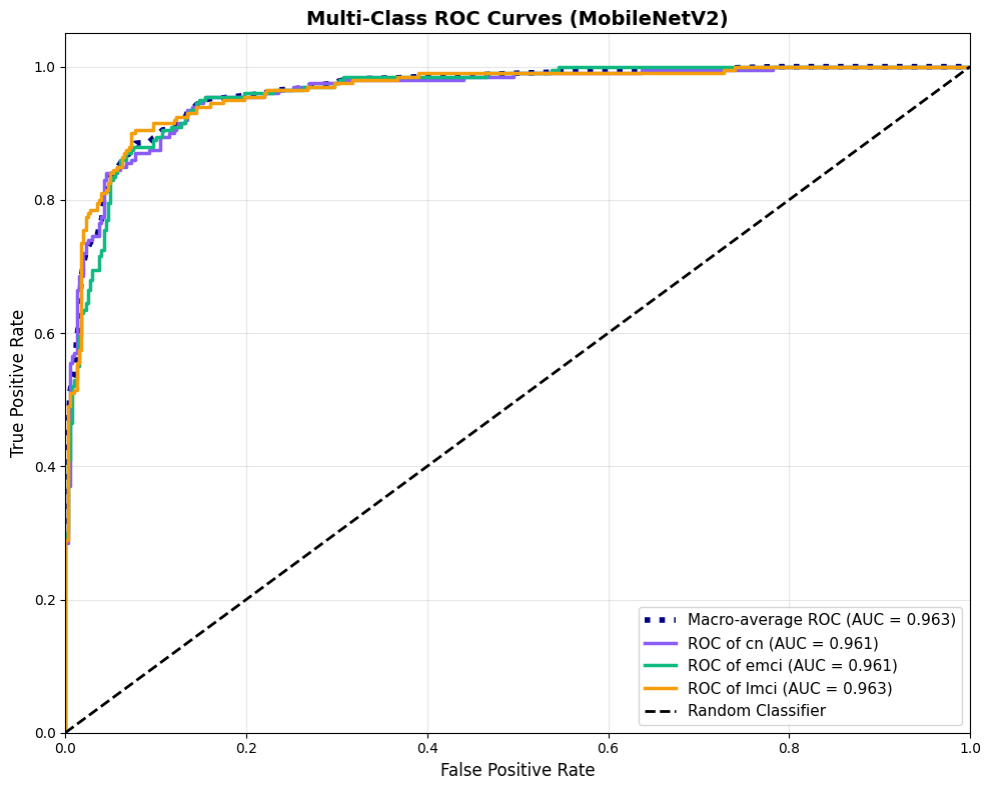
ROC curves with AUC values above 0.95, for MobileNetV2 base configuration.

**Figure 8:**
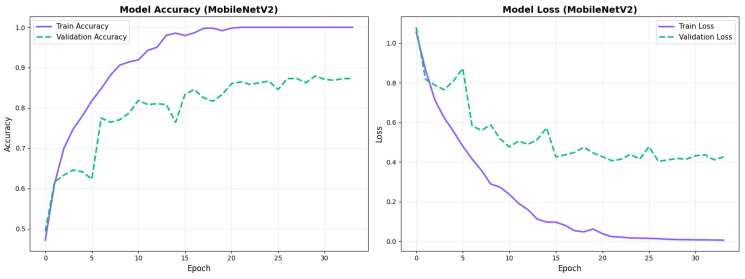
Training curves for MobileNetV2 original configuration showing stable convergence.

**Figure 9:**
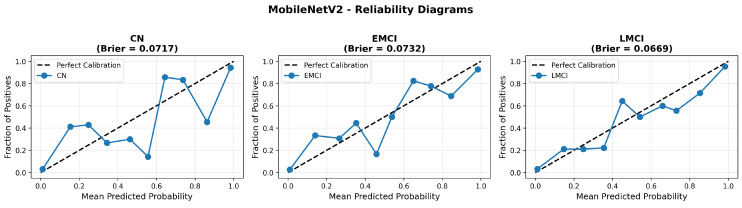
Reliability diagrams for MobileNetV2 original configuration across all three diagnostic classes.

MobileNetV2 achieved consistent performance with 0.86 accuracy in the original configuration and 0.865 with augmentation. The original configuration showed balanced performance across classes with EMCI precision (0.84) and recall (0.88), while augmentation maintained similar EMCI performance (0.85 precision, 0.86 recall). The confusion matrix (Figure 6) shows a strong diagonal classification with primary confusion between the CN and EMCI classes. ROC analysis (Figure 7) demonstrates robust discriminative performance with AUC values above 0.90 for all pairs of classes. Figure 8 shows the MobileNetV2 training curves with the original configuration highlighting the training and validation accuracy, as well as the training and validation loss. These three figures depict the measurements for MobileNetV2 in the original configuration. MobileNetV2 original configuration showed reasonably well-calibrated probability estimates with an average Brier score of 0.0706 (CN: 0.0717, EMCI: 0.0732, LMCI: 0.0669), though some deviation from the diagonal in mid-range confidence bins suggested room for improvement (Figure 9).

**Figure 10:**
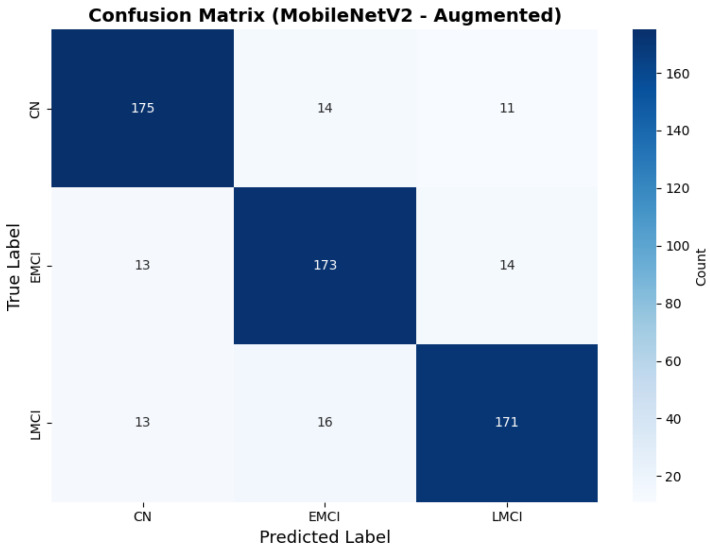
Confusion matrix with improved class balance for MobileNetV2 augmented configuration.

**Figure 11:**
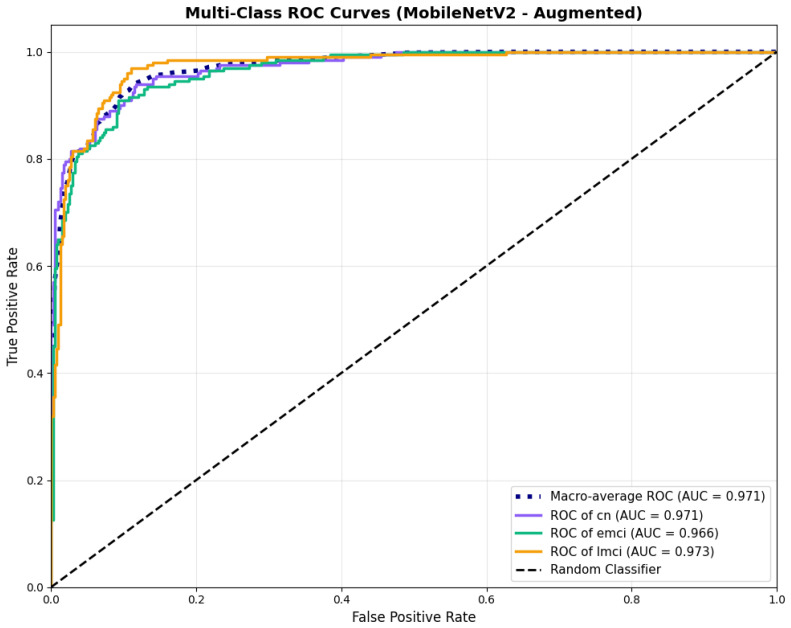
ROC curves showing enhanced performance, for MobileNetV2 augmented configuration.

**Figure 12:**
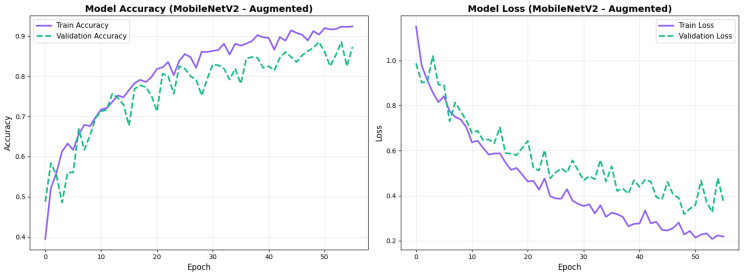
Training curves for MobileNetV2 augmented configuration with smoother validation.

**Figure 13:**
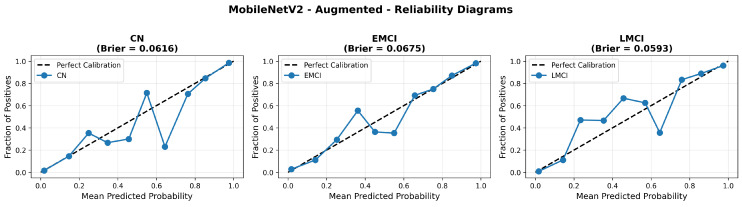
Reliability diagrams for EfficientNetV2B0 original configuration showing baseline calibration performance.

Similarly, the confusion matrix (Figure 10) shows a strong diagonal classification with primary confusion between the CN and EMCI classes. ROC analysis (Figure 11) demonstrates robust discriminative performance with AUC values above 0.90 for all pairs of classes. Figure 12 shows the MobileNetV2 training curves with the augmented configuration highlighting the training and validation accuracy, as well as the training and validation loss. These three figures depict the measurements for MobileNetV2 in the augmented configuration. Data augmentation substantially improved calibration to an average Brier score of 0.0628, with probability estimates more closely tracking the ideal diagonal across all classes, particularly for LMCI (Brier: 0.0593) and CN (Brier: 0.0616), confirming the dual benefit of augmentation for both discrimination and probability reliability (Figure 13). Data augmentation maintained balanced performance across classes with CN recall (0.88), EMCI recall (0.86), and LMCI recall (0.85).

#### 4.3.2. EfficientNetV2B0 Results

EfficientNetV2B0 demonstrated superior overall performance. The original configuration achieved balanced performance with CN precision (0.90) and recall (0.88), with strong EMCI recall (0.90). Augmentation improved CN precision to 0.93 while maintaining good recall (0.83), and enhanced EMCI recall to 0.93.

**Figure 14:**
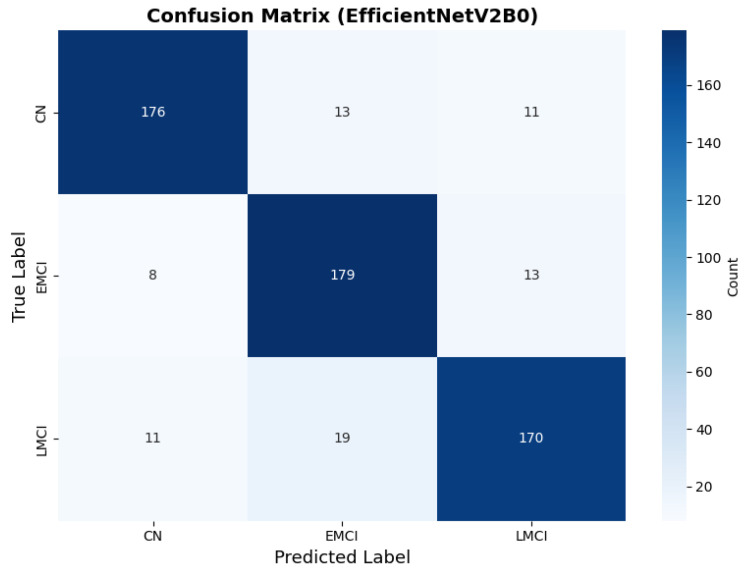
Confusion matrix with high CN specificity for EfficientNetV2B0 original configuration.

**Figure 15:**
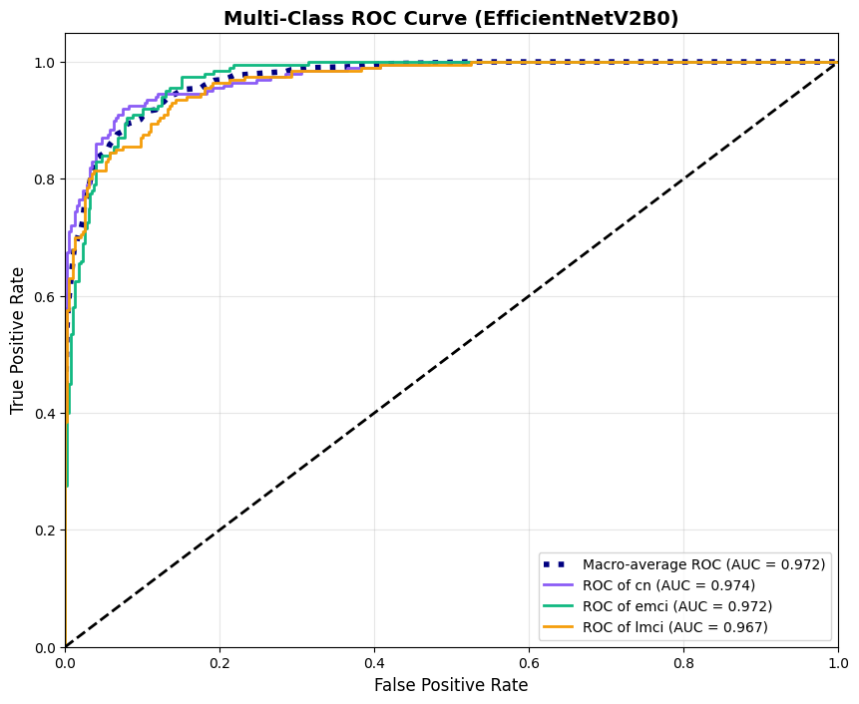
ROC curves with AUC values exceeding 0.95, for EfficientNetV2B0 original configuration.

**Figure 16:**
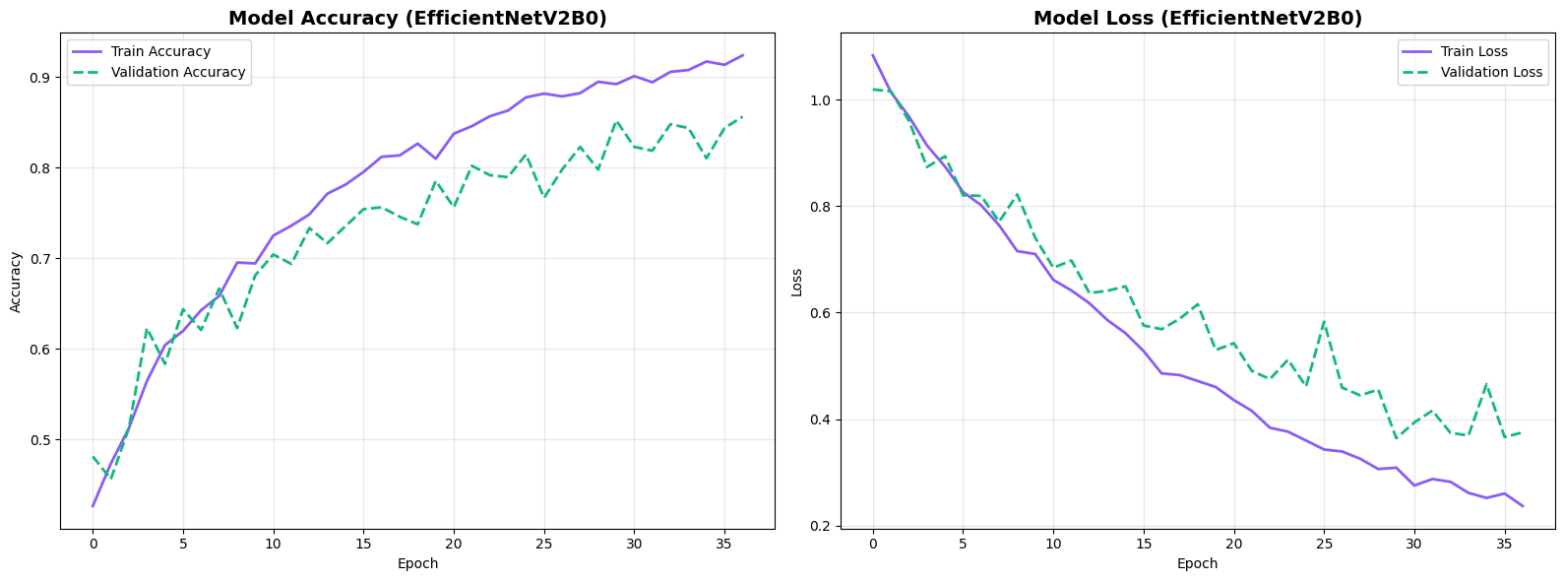
Training curves for EfficientNetV2B0’s original configuration with rapid initial convergence.

**Figure 17:**
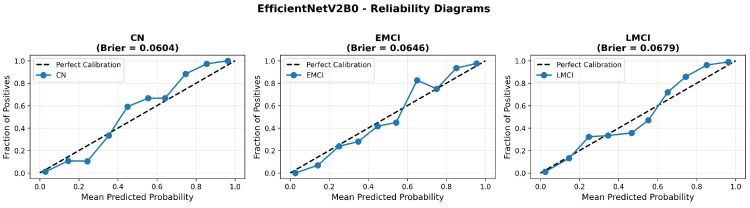
Reliability diagrams for EfficientNetV2B0’s original configuration showing baseline calibration performance.

**Figure 18:**
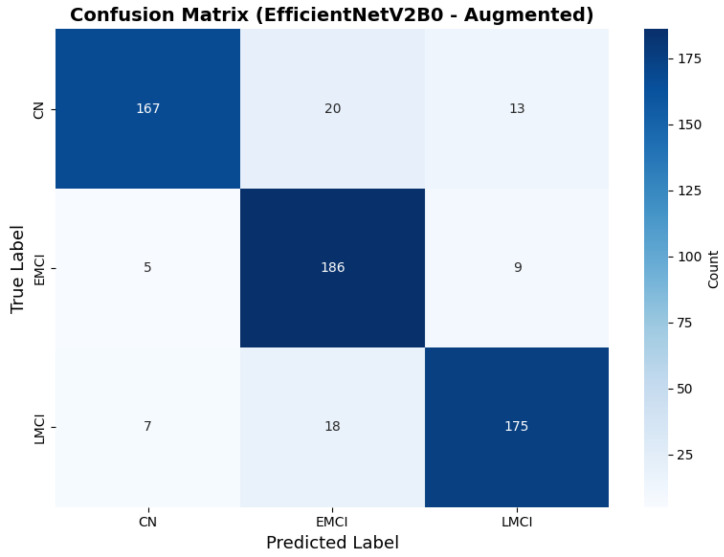
Confusion matrix showing optimal class balance, for EfficientNetV2B0 augmented configuration.

**Figure 19:**
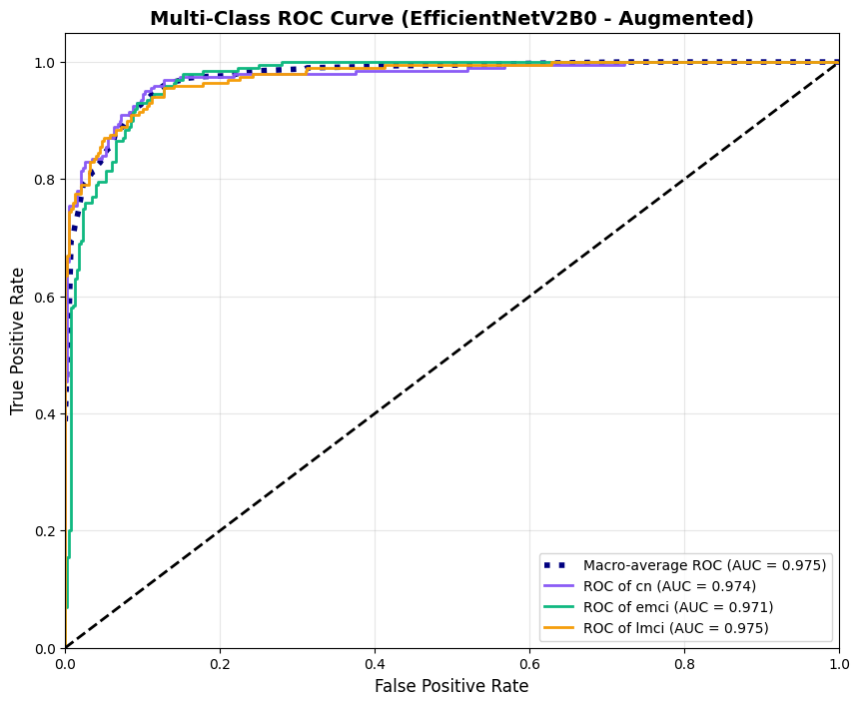
ROC curves with superior discriminative performance, for EfficientNetV2B0’s augmented configuration.

**Figure 20:**
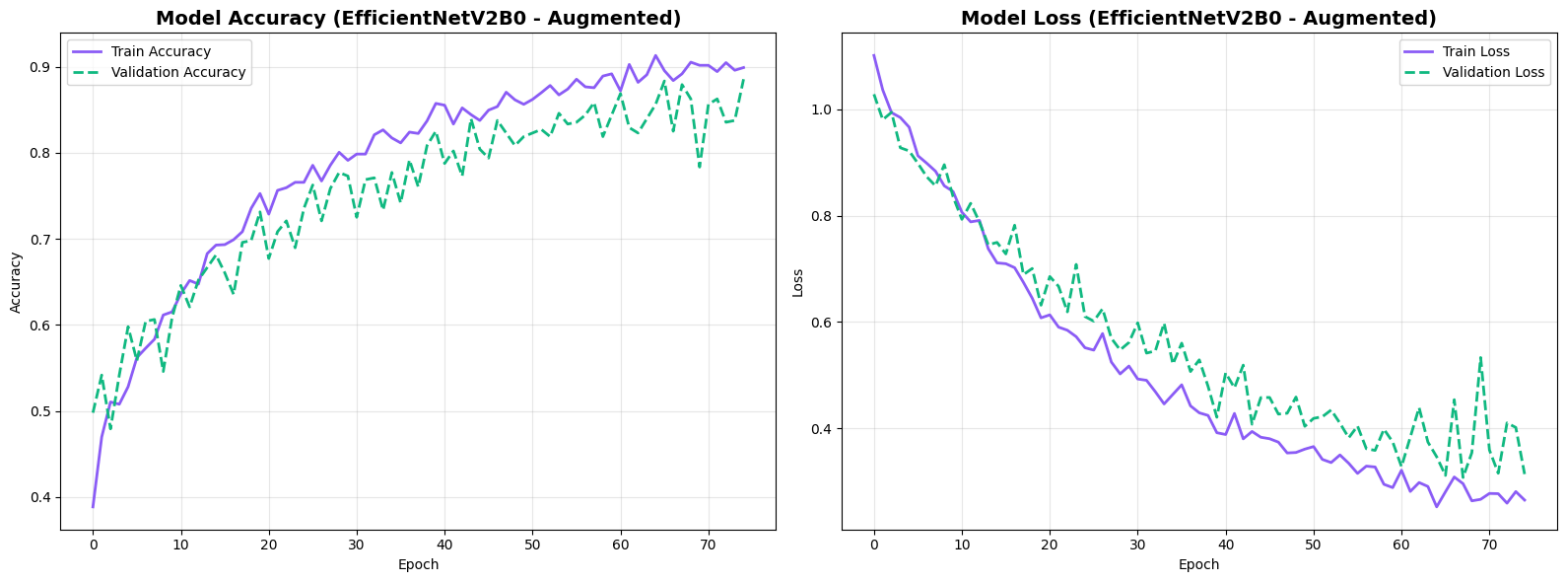
Training curves for EfficientNetV2B0’s augmented configuration showing stable convergence.

**Figure 21:**
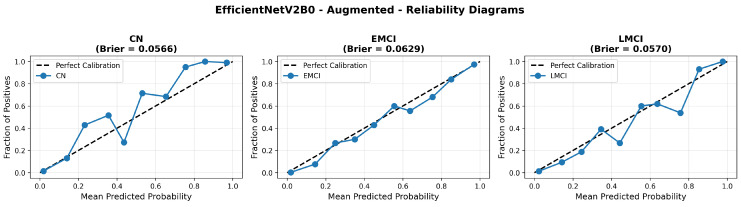
Reliability diagrams for EfficientNetV2B0’s augmented configuration achieving optimal calibration.

The confusion matrix (Figure 14) shows a strong diagonal classification with primary confusion between the LMCI and EMCI classes. ROC analysis (Figure 15) demonstrates robust discriminative performance with AUC values above 0.90 for all pairs of classes. Figure 16 shows the EfficientNetV2B0 training curves with the original configuration that highlights the accuracy of the training and the validation, as well as the loss of training and validation. These three figures depict the measurements for EfficientNetV2B0 in the original configuration. EfficientNetV2B0 demonstrated strong baseline calibration in the original configuration (average Brier: 0.0643), with excellent calibration for CN (Brier: 0.0604) and competitive calibration for EMCI (Brier: 0.0646) and LMCI (Brier: 0.0679) (Figure 17). Similarly, Figure 18 and Figure 19 show the confusion matrix and ROC for the augmented configuration. Figure 20 depicts the accuracy and loss curves for training and validation for each epoch. The augmented configuration achieved optimal calibration (average Brier: 0.0588), the best among all tested architectures, showing exceptional calibration uniformly across all classes: CN (Brier: 0.0566), EMCI (Brier: 0.0629), and LMCI (Brier: 0.0570), with predicted probabilities demonstrating near-perfect adherence to observed frequencies across all confidence bins (Figure 21).

The augmented configuration achieved the highest overall performance (0.88 accuracy) with strong performance across all classes: CN (0.93 precision, 0.83 recall), EMCI (0.83 precision, 0.93 recall), and LMCI (0.89 precision, 0.88 recall).

#### 4.3.3. Training Dynamics and Convergence Patterns

The training dynamics revealed distinct convergence patterns between the two architectures. EfficientNetV2B0 demonstrated extended convergence periods, requiring 30–68 epochs to reach optimal performance. This extended learning phase contrasts with MobileNetV2, which converged more rapidly at 27–49 epochs, suggesting EfficientNetV2B0’s capacity for continued feature refinement.

The prolonged convergence in EfficientNetV2B0 can be attributed to its compound scaling mechanism and more complex Fused-MBConv blocks, which create a richer optimization landscape requiring more training iterations to reach its full potential. The model’s built-in regularization techniques, including adaptive depth-wise convolution and progressive learning strategies, likely contributed to this gradual but stable improvement pattern. This architectural complexity enables the model to capture more nuanced neuroanatomical patterns essential for distinguishing subtle early-stage Alzheimer’s conditions (EMCI vs. LMCI).

From a practical perspective, these findings indicate that pre-convergence early stopping would substantially undermine EfficientNetV2B0’s performance potential. The extended learning trajectory correlates with the model’s superior final performance (88.0% accuracy vs. 86.5% for MobileNetV2), suggesting that the additional training epochs contributed meaningfully to feature optimization for fine-grained classification tasks. Future implementations should employ patience-based stopping criteria with extended tolerance periods (≥30 epochs) rather than fixed epoch budgets to fully leverage the architecture’s learning capacity.

#### 4.3.4. Testing on Other Architecture Variants

**Figure 22:**
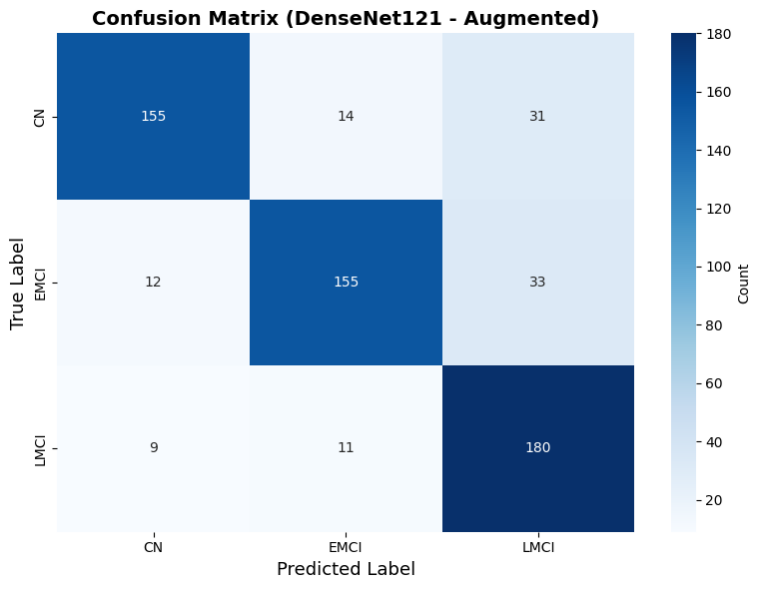
Confusion for DenseNet121 augmented configuration.

**Figure 23:**
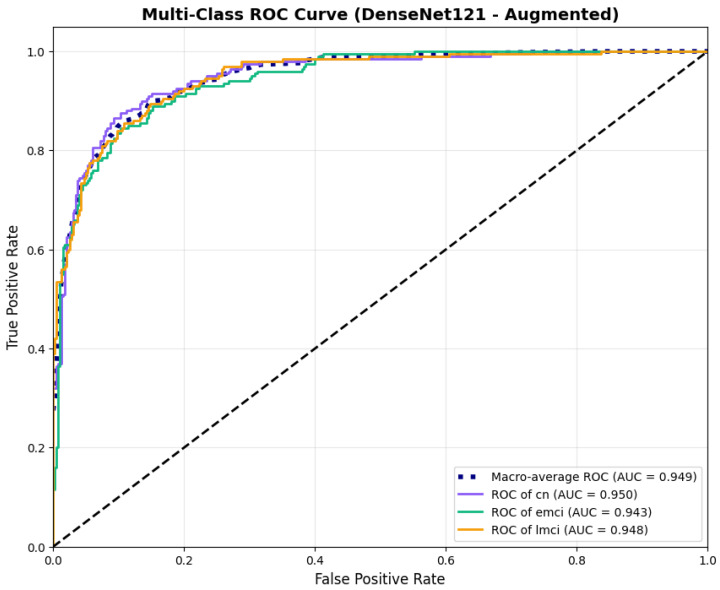
ROC curves for DenseNet121 augmented configuration.

**Figure 24:**
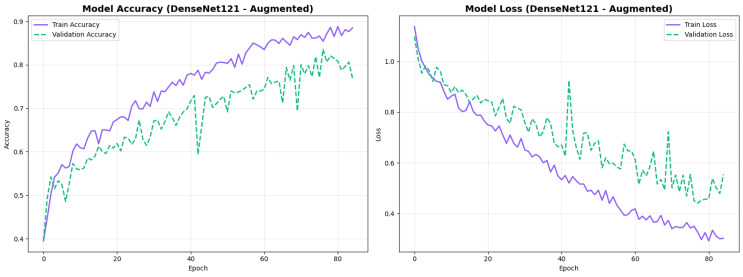
Training curves for DenseNet121 augmented configuration.

**Figure 25:**
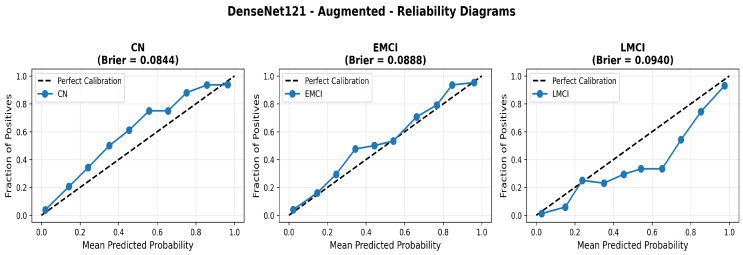
Reliability diagrams for DenseNet121 augmented configuration showing calibration limitations.

To address the robustness of the proposed methodology, we have tested the other variants of architectures, such as DenseNet121 [64]. The results were found using the base model of DenseNet121, and they are depicted in the following figures. Figure 22 shows the confusion matrix and Figure 23 shows the ROC curves for DenseNet121. The training curves, including the model accuracy and model loss, are shown in Figure 24. In contrast, DenseNet121 augmented configuration exhibited substantially poorer calibration (average Brier: 0.0891) compared to both lightweight architectures, with pronounced deviation from the ideal diagonal across all classes: CN (Brier: 0.0844), EMCI (Brier: 0.0888), and LMCI (Brier: 0.0940). The systematic underestimation of predicted probabilities in lower-confidence bins and overestimation in higher bins suggests the deeper architecture produces miscalibrated predictions despite reasonable discrimination performance (AUC: 0.947), highlighting a key limitation of heavyweight architectures for this task (Figure 25).

#### 4.3.5. Cross-Validation Analysis

To address concerns about single train–test split bias and ensure reliable performance estimation, we conducted comprehensive 5-fold stratified cross-validation as our primary evaluation methodology. Each model configuration was trained and evaluated independently on five different data partitions, providing robust evidence of generalizability free from arbitrary split artifacts.

The complete dataset of 3000 slices was partitioned into five stratified folds using scikit-learn’s StratifiedKFold implementation, ensuring balanced class distribution (33.3% per class) across all folds. For each fold, 2400 samples (800 per class) served as the training set, and 600 samples (200 per class) as the validation set. Models were trained independently on each fold using identical hyperparameters (Table 6), with early stopping based on validation loss (patience = seven epochs). This protocol yielded five independent performance estimates per model configuration, totaling 25 trained models across all architectures.

Table 12 presents the aggregated performance across all five folds for each model configuration.

The low standard deviations across all folds (0.01–0.025) show consistent performance regardless of data partitioning, validating model robustness. EfficientNetV2B0 with augmentation achieved the highest mean accuracy (88.0%) with excellent stability (std = 1.0%), while DenseNet121 showed greater variance (std = 2.52%) with lower overall performance (81.0%).

Table 13 details the class-specific precision, recall, and F1-scores averaged across all five folds, demonstrating balanced performance across diagnostic categories.

All models maintained balanced performance across the three diagnostic categories, with no single class showing systematic under-performance. EfficientNetV2B0 augmented demonstrated particularly strong EMCI recall (0.93 ± 0.03).

To demonstrate the consistency of our cross-validation results, Table 14 presents individual fold performance for our best model (EfficientNetV2B0 Augmented), showing the narrow performance range that confirms result reliability.

The narrow accuracy range (86.5–89.5%) across all five folds confirms that model performance is not dependent on specific data partitioning. Fold-wise analysis also revealed architecture-specific learning patterns: MobileNetV2 configurations showed consistent convergence (std = 5–8 epochs), while EfficientNetV2B0 exhibited larger convergence variability (std = 12 epochs), reflecting its more complex optimization landscape from compound scaling that ultimately yields superior performance.

#### 4.3.6. Summary of the Model Results

The analysis of computational efficiency revealed distinct characteristics between architectures. During training, MobileNetV2 demonstrated faster step times (70–240 ms/step) compared to EfficientNetV2B0 (240–300 ms/step). However, EfficientNetV2B0’s marginally increased training time is justified by its superior performance across all metrics: +1.5% absolute accuracy, +0.3% macro AUC improvement, and −6.4% Brier score reduction compared to MobileNetV2 augmented. Both architectures maintained practical inference speeds suitable for real-time clinical deployment while achieving excellent calibration (Brier≤0.063).

Data augmentation strategies exhibited architecture-dependent effects across discrimination and calibration metrics. For EfficientNetV2B0, augmentation improved accuracy from 0.8750 to 0.8800 (+0.57%), enhanced macro AUC from 0.971 to 0.973 (+0.21%), and substantially improved calibration with a Brier score reduction from 0.0643 to 0.0588 (−8.6%). The reliability diagrams (Figure 17 and Figure 21) demonstrate that augmentation resulted in predicted probabilities that more uniformly track observed frequencies across all confidence bins, particularly improving high-confidence predictions. MobileNetV2 exhibited similar patterns: accuracy improved from 0.86 to 0.8650 (+0.58%), macro AUC increased from 0.962 to 0.970 (+0.83%), and Brier score decreased from 0.0706 to 0.0628 (−11.0%). Notably, both lightweight architectures substantially outperformed DenseNet121 across all metrics: accuracy advantage of +4.8–6.3%, macro AUC improvement of +2.3–2.6%, and dramatic calibration superiority with Brier scores 29.5–34.0% lower. These findings validate that efficient architectures leveraging compound scaling (EfficientNetV2B0) and depthwise separable convolutions (MobileNetV2) achieve superior accuracy–efficiency–calibration trade-offs compared to traditional deep architectures, establishing EfficientNetV2B0 as the optimal model for clinical deployment based on its balanced excellence across all evaluation dimensions.

### 4.4. Statistical Analysis of Inter-Model Agreement

To validate the statistical significance of observed performance differences and assess whether different architectures learn similar underlying patterns, we conducted chi-square tests of independence on model predictions. The chi-square test was selected as the appropriate statistical method because it evaluates the independence of categorical predictions between models, making it well-suited for multi-class classification scenarios where we need to determine whether different architectures make significantly correlated or independent predictions on the same test instances.

#### 4.4.1. Methodology and Rationale

The chi-square test of independence examines whether two models’ predictions are statistically independent or show significant agreement patterns. This test is well-suited for multi-class classification scenarios as it directly evaluates the relationship between categorical predictions made by different models on identical test instances.

For each pair of models (MobileNetV2 vs. EfficientNetV2B0, MobileNetV2 vs. DenseNet121, EfficientNetV2B0 vs. DenseNet121), we constructed a contingency table where each cell represents the frequency of instances jointly classified into specific class combinations by both models. The test statistic is computed as follows:

(1) $$\chi^{2}=\sum_{i,j}\frac{{\left(O_{ij}-E_{ij}\right)}^{2}}{E_{ij}}$$

where $O_{ij}$ represents the observed frequency in cell (i,j) and $E_{ij}$ represents the expected frequency under the null hypothesis of independence. The null hypothesis ($H_{0}$) assumes that the two models make independent predictions (i.e., knowing one model’s prediction provides no information about the other’s prediction), while the alternative hypothesis ($H_{1}$) suggests that the models show significant agreement in their classification patterns. Large chi-square values with correspondingly small *p*-values indicate that the models’ predictions are significantly correlated, rejecting the independence assumption.

#### 4.4.2. Pairwise Agreement Analysis

We evaluated pairwise agreement between three augmented model configurations: MobileNetV2, EfficientNetV2B0, and DenseNet121. For each pair, we calculated the chi-square statistic, *p*-value, degrees of freedom, and overall agreement percentage. The results are presented in Table 15.

All three model pairs demonstrated statistically significant agreement patterns (p<0.001), rejecting the null hypothesis of independence. This indicates that the models converge on similar classification decisions for a substantial proportion of test instances, despite architectural differences. The agreement rates ranged from 73.83% (MobileNetV2-DenseNet121) to 78.00% (MobileNetV2-EfficientNetV2B0), suggesting that all three architectures learn similar underlying patterns from the neuroimaging data. The extremely low *p*-values (far below the conventional α=0.05 threshold) provide strong evidence that the performance differences observed between architectures are not attributable to random variation.

#### 4.4.3. Contingency Table Analysis

To understand the specific patterns of agreement and disagreement, we examined the contingency tables for each model pair. These tables reveal where models agree most strongly and where they exhibit systematic differences in classification behavior.

The MobileNetV2-EfficientNetV2B0 comparison (Table 16) shows strong diagonal agreement, with both models correctly identifying 138 CN cases, 173 EMCI cases, and 157 LMCI cases in concordance. The primary disagreements occur in the EMCI classification, where MobileNetV2 classified 26 cases as CN that EfficientNetV2B0 identified as EMCI, and 25 cases as LMCI that EfficientNetV2B0 classified as EMCI. This pattern suggests that EfficientNetV2B0 may be more confident in identifying the intermediate EMCI stage, while MobileNetV2 exhibits more uncertainty between adjacent categories.

The MobileNetV2-DenseNet121 comparison (Table 17) reveals the lowest agreement rate (73.83%) among the three pairs. DenseNet121 shows a notable tendency to classify cases as LMCI (244 total LMCI predictions compared to 201 by MobileNetV2), suggesting that this architecture may be more sensitive to advanced neurodegenerative features. The disagreement is particularly pronounced for cases that MobileNetV2 classified as CN or EMCI but DenseNet121 classified as LMCI (38 and 42 instances, respectively), indicating systematic differences in how these architectures weight early- versus late-stage pathological features.

The EfficientNetV2B0-DenseNet121 comparison (Table 18) shows 77.33% agreement, intermediate between the other two pairs. Similar to the MobileNetV2-DenseNet121 comparison, DenseNet121 again demonstrates a bias toward LMCI classification. The most substantial disagreement occurs when EfficientNetV2B0 predicts EMCI but DenseNet121 predicts LMCI (49 instances), suggesting that DenseNet121’s deeper architecture may capture more subtle signs of disease progression that lead it to classify cases at more advanced stages.

#### 4.4.4. Interpretation of Agreement Patterns

The statistical validation shows that while all architectures demonstrate substantial agreement (>73%), the systematic differences in their disagreements reflect architectural characteristics rather than random variation. The high chi-square statistics ($\chi^{2}>450$) with extremely low *p*-values confirm that observed performance differences reflect genuine architectural characteristics rather than statistical noise or random variation.

EfficientNetV2B0 exhibited the most balanced classification across all three diagnostic categories, while DenseNet121 showed a consistent bias toward identifying more advanced disease stages. These patterns, which are systematic rather than random, suggest that different architectures capture complementary aspects of AD pathophysiology. This observation supports our selection of EfficientNetV2B0 as the optimal model for clinical deployment, as its balanced sensitivity across disease stages is particularly valuable for early detection scenarios where distinguishing between CN, EMCI, and LMCI is most challenging.

### 4.5. Explainability Analysis

To validate that our models learn clinically meaningful features, we applied a multi-scale attribution pipeline combining Grad-CAM++ and Guided Grad-CAM++. This approach generates anatomically grounded explanations by synthesizing coarse-grained regional importance with fine-grained pixel-level attributions.

#### 4.5.1. Explainability Pipeline

**Figure 26:**
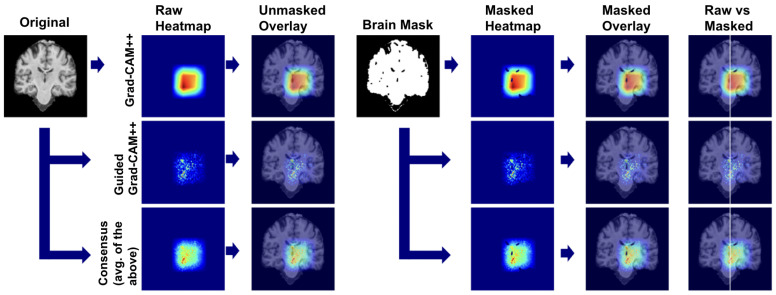
Multi-scale explainability pipeline combining coarse- and fine-grained attribution methods.

We applied our attribution methodology through a systematic multi-stage process (Figure 26). First, we extracted class-discriminative heatmaps using Grad-CAM++ from the final convolutional layer, providing broad anatomical localization. Second, we applied Guided Grad-CAM++ to capture fine-grained features within relevant regions. Third, we generated consensus maps by averaging the normalized attribution maps from both methods, creating a unified visualization that highlights features consistently important at both coarse and fine scales. Finally, we applied brain masking to eliminate background artifacts and focus interpretation on relevant neuroanatomy.

We used this pipeline to leverage the complementary strengths of each method: Grad-CAM++ provides robust regional localization while Guided Grad-CAM++ captures detailed textural patterns. The consensus map combines these perspectives, reducing individual method limitations while preserving anatomically consistent features across both attribution scales.

#### 4.5.2. Class-Specific Attribution Patterns

The following explainability visualizations were generated using our best-performing model, EfficientNetV2B0, which was trained on augmented data.

##### Cognitively Normal (CN) Classifications

**Figure 27:**
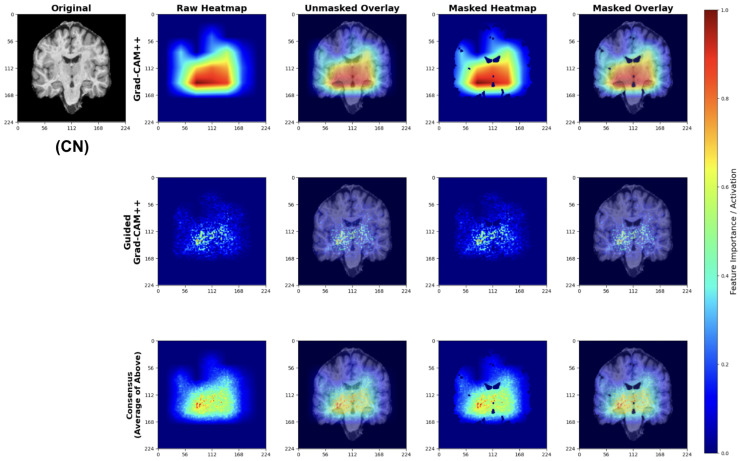
Explainability results for CN classification showing focus on preserved brain structures.

For CN predictions, our models consistently focus on healthy brain morphology patterns (Figure 27). The Grad-CAM++ heatmaps highlight normal-sized ventricular spaces and preserved hippocampal regions, while Guided Grad-CAM++ reveals fine-scale tissue integrity patterns. The consensus maps demonstrate model attention to compact ventricular spaces and intact medial temporal structures, indicating the models correctly identify hallmarks of healthy brain architecture.

The attribution patterns align with clinical expectations, showing minimal attention to regions typically affected by early neurodegeneration.

##### Early Mild Cognitive Impairment (EMCI) Classifications

**Figure 28:**
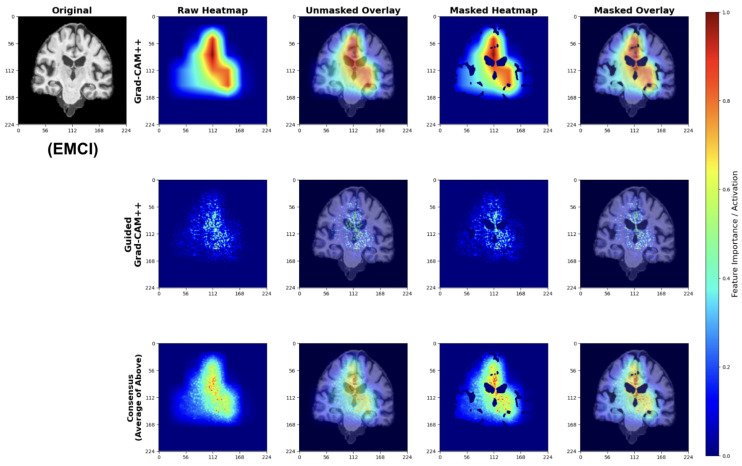
Explainability results for EMCI classification highlighting early pathological changes.

EMCI explanations reveal model sensitivity to subtle early-stage pathological changes (Figure 28). The Grad-CAM++ analysis highlights both initial ventricular enlargement and early hippocampal alterations, while Guided Grad-CAM++ captures initial tissue changes in medial temporal regions. The consensus maps effectively combine both perspectives, showing focused attention on regions exhibiting the earliest measurable structural modifications.

These patterns indicate the model successfully learns to detect subtle structural changes preceding more advanced cognitive decline.

##### Late Mild Cognitive Impairment (LMCI) Classifications

**Figure 29:**
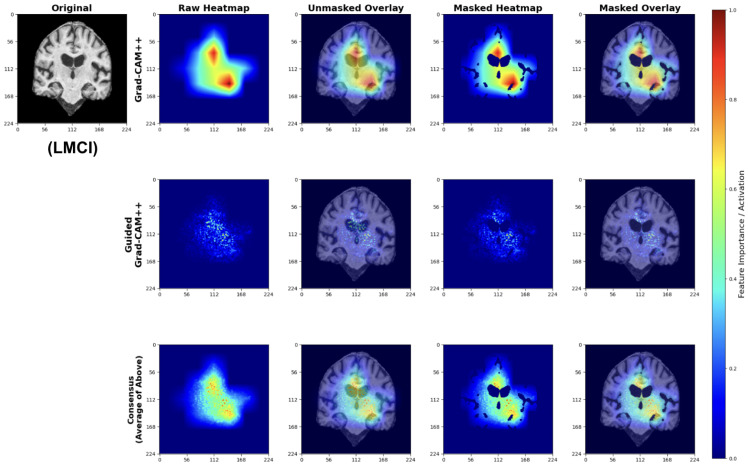
Explainability results for LMCI classification showing advanced neurodegenerative patterns.

LMCI attributions show pronounced attention to advanced neurodegenerative patterns (Figure 29). The Grad-CAM++ maps highlight both significant ventricular enlargement and pronounced hippocampal atrophy, while Guided GradCAM++ captures widespread tissue loss patterns. The consensus maps demonstrate unified model focus on multiple indicators of advanced neurodegeneration, including expanded ventricular spaces and substantial medial temporal lobe volume reduction.

These attribution patterns align with established neuropathological progression, validating model learning of clinically relevant features.

#### 4.5.3. Anatomical Validation

The attribution maps demonstrate strong correspondence with known AD pathophysiology. Across all classes, models show appropriate attention to medial temporal structures. The progressive increase in ventricular and sulcal attention from CN to LMCI classifications reflects the expected pattern of advancing neurodegeneration [65].

Importantly, the model avoids false correlations with non-anatomical features, as evidenced by the brain-masked attributions focusing exclusively on neuroanatomically relevant regions. The consensus mapping approach successfully reduces method-specific artifacts while preserving consistent anatomical focus.

While our explainability analysis using Grad-CAM++ and Guided Grad-CAM++ qualitatively highlights anatomically meaningful regions such as the hippocampus and ventricular areas—which are known to be affected in early Alzheimer’s disease—we acknowledge that a quantitative validation of these attributions against structural biomarkers (e.g., hippocampal volume loss) was not performed in the present study. Nonetheless, the spatial consistency of the attention maps across multiple subjects and their correspondence with well-documented neurodegenerative regions suggest that the models are learning disease-relevant representations rather than dataset-specific artifacts. Future work will include quantitative overlap analysis with standard neuroanatomical atlases and correlation with volumetric measures derived from tools such as FreeSurfer v8.0.0 to objectively validate the biological plausibility of the model’s attributions.

#### 4.5.4. Clinical Interpretability

Our explainability analysis demonstrates that the lightweight models identify anatomically relevant brain regions consistent with established AD pathophysiology. The attribution patterns show a logical progression: preserved hippocampal structures in CN cases, subtle medial temporal changes in EMCI, and pronounced ventricular enlargement with widespread atrophy in LMCI. This progression aligns with documented patterns of neurodegeneration [26,27].

The multi-scale attribution pipeline successfully combines coarse regional localization with fine-grained feature analysis. Grad-CAM++ highlights broad anatomical regions affected by disease progression, while Guided Grad-CAM++ reveals specific textural changes within these areas. The consensus maps synthesize both perspectives, reducing individual method limitations while maintaining focus on clinically meaningful structures.

Importantly, our models avoid attention to non-anatomical artifacts, as evidenced by brain-masked visualizations that focus exclusively on neuroanatomically relevant regions. This anatomical specificity suggests the models learn disease-relevant features rather than dataset biases.

The interpretability framework is designed to meet the practical demands of clinical deployment. Lightweight architectures must strike a balance between computational efficiency and explanation quality, and our consensus approach achieves transparent decision-making while preserving inference speeds suitable for everyday clinical workflows. The anatomically grounded visualizations allow clinicians to cross-check model predictions with their own expertise, potentially enhancing diagnostic confidence in resource-limited environments.

This interpretability validation supports the broader goal of deploying accessible AI tools for early AD detection. By demonstrating that our efficient models focus on clinically established disease markers, we provide evidence that computational efficiency need not compromise clinical relevance. The transparent attribution mechanisms facilitate clinician understanding and trust, essential requirements for successful integration into diagnostic workflows.

## 5. Discussion

In this section, we discuss the advantage of the models applied in this research to strengthen the clinical implications as well as translational impact. The development of neuroimaging analysis tools provides the ground to utilize the models by the clinicians.

### 5.1. Translational and Clinical Implications

To address a critical methodological concern of single train-test split bias, we employed a 5-fold stratified cross-validation. Our evaluation across five independent splits provides robust evidence of model generalizability and performance stability. The consistently low standard deviations across all folds (0.01–0.025 for accuracy metrics) demonstrate that our findings are reproducible and not artifacts of favorable data splitting. The cross-validation results also revealed architecture-specific patterns, such as higher convergence variability (std = 12 epochs) with EfficientNetV2B0 against MobileNetV2’s consistency (std = 5–8 epochs), that would not be apparent from single-split evaluation and inform deployment decisions.

The translational potential of this study lies in the bridge between advanced AI methods and practical clinical utility. By employing lightweight architectures such as MobileNetV2 and EfficientNetV2B0, our framework demonstrates that accurate early detection of Alzheimer’s disease can be achieved using standard structural MRI scans. Inference benchmarking validates this claim quantitatively. Our best-performing model (EfficientNetV2B0 augmented) processes a complete 30-slice study in 10.4 s on standard CPU hardware, achieving a throughput of 347 studies per hour. MobileNetV2, while slightly less accurate, offers even faster processing at 5.2 s per study (687 studies/hour) with a minimal 11.1 MB model footprint. These performance characteristics enable deployment on commodity hardware without GPU acceleration, addressing the critical barrier of computational resource availability in underserved clinical environments. This positions the proposed approach as a viable decision-support tool for low-resource or community-based healthcare settings where access to neuroradiology expertise and advanced imaging modalities is limited. The integration of explainable AI techniques, namely Grad-CAM++ and Guided Grad-CAM++, further enhances clinical interpretability by providing anatomically meaningful visualizations that correspond to disease-relevant regions such as the hippocampus and medial temporal lobes. These features can assist radiologists in verifying model outputs, promoting trust and transparency in AI-assisted diagnosis. Future work will focus on multi-center validation, incorporation of 3D volumetric and multi-modal MRI sequences, and clinician-in-the-loop testing to facilitate regulatory acceptance and seamless integration into clinical workflows. The following subsection introduces the neuroimaging analysis tool that we developed, along with the implementation steps, pre-processing integrations as described in the methodology section and deployment consideration made during the implementaion process.

Recent neuroimaging research has increasingly recognized ventricular enlargement as a sensitive and quantifiable biomarker of early Alzheimer’s disease progression. Ventricular expansion reflects underlying cortical and subcortical atrophy—particularly in the medial temporal and hippocampal regions—and may precede measurable cognitive decline. Our explainability analysis, which consistently highlighted periventricular regions across EMCI and LMCI groups, aligns with these findings. Notably, recent studies have demonstrated that longitudinal ventricular volume changes correlate strongly with cognitive impairment severity and conversion from mild cognitive impairment to Alzheimer’s disease [66], and that ventricular morphology provides complementary predictive value to hippocampal atrophy for early diagnosis [67]. These converging lines of evidence reinforce the biological plausibility of our model’s attention patterns and suggest that ventricular enlargement, alongside hippocampal degeneration, could serve as a robust imaging marker for early-stage AD detection in clinical workflows.

#### Neuroimaging Analysis Tool

To demonstrate practical deployment of our lightweight explainable framework, we developed a neuroimaging analysis application that integrates our trained models with real-time explainability visualization for clinical evaluation workflows.

The tool consists of two main components: a comprehensive slice viewer for medical image navigation and annotation, and a specialized XAI analysis module for model inference and explainability visualization. The slice viewer implements medical imaging functionality including multi-planar viewing, window/level adjustment, measurement capabilities, and annotation tools using Python 3.11.3 with SimpleITK 2.5.2 for medical image handling and TensorFlow for neural network execution. The analysis module integrates our best model, the EfficientNetV2B0 model trained on augmented data with configurable layer selection for different explainability methods. The system includes dynamic layer configuration interfaces that automatically detect convolutional layers in loaded models and enable users to specify attribution layers for Grad-CAM++, Guided Grad-CAM++, and consensus attribution maps across different architectures.

To demonstrate practical deployment of our lightweight explainable framework, we developed a neuroimaging analysis application that integrates our trained models with real-time explainability visualization for clinical evaluation workflows.

**Figure 30:**
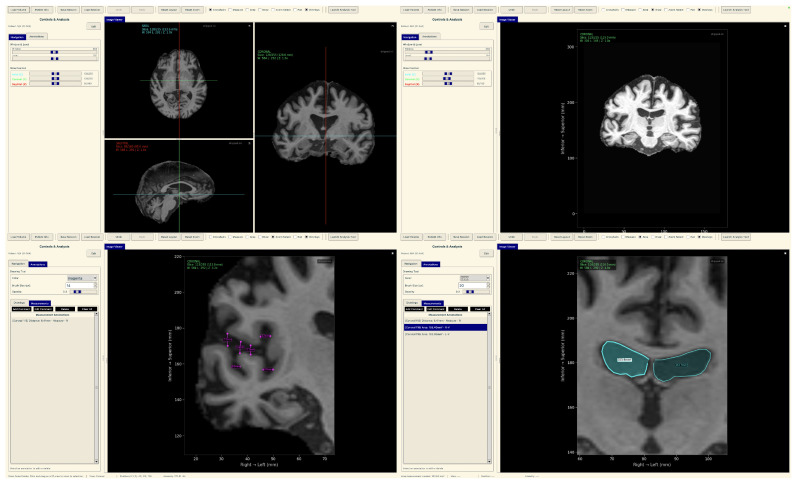
Neuroimaging slice viewer interface demonstrating MRI visualization capabilities.

**Figure 31:**
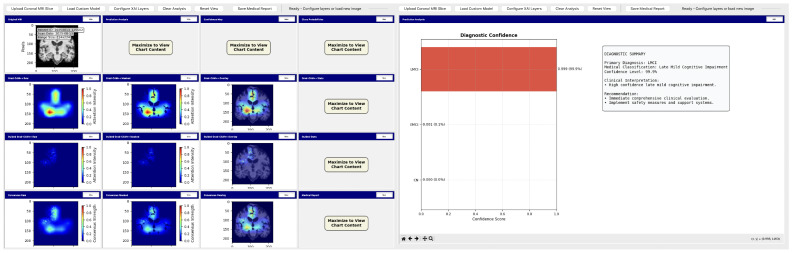
Analysis tool interface demonstrating AI explainability features.

The tool consists of two main components: a comprehensive slice viewer for medical image navigation and annotation (Figure 30), and a specialized XAI analysis module for model inference and explainability visualization (Figure 31). The slice viewer implements medical imaging functionality including multi-planar viewing, window/level adjustment, measurement capabilities, and annotation tools using Python with SimpleITK for medical image handling and TensorFlow for neural network execution.

The analysis tool implements a simplified preprocessing pipeline suitable for demonstration purposes, including basic cropping and resizing to the required input dimensions 224×224. The complete preprocessing pipeline described in the data set and preprocessing (Section 3.1) should be applied before using this tool.

The application maintains computational efficiency suitable for standard clinical hardware while providing session management, annotation capabilities, and structured reporting. The modular architecture supports both clinical demonstration and research validation, enabling comparative studies of lightweight architectures within a consistent framework. The complete implementation serves as a proof-of-concept for the deployment of accessible AI tools in routine clinical settings.

### 5.2. Addressing Generalizability and External Validation

While the current study is based on a relatively modest cohort of 102 subjects from the ADNI database, this dataset offers several advantages that justify its use for developing and benchmarking early Alzheimer’s detection models. ADNI is one of the most rigorously curated and quality-controlled neuroimaging repositories, providing standardized MRI acquisition protocols, consistent diagnostic labeling, and extensive clinical metadata. These features ensure methodological reliability and comparability with prior studies, as summarized in Table 2. Nevertheless, we acknowledge that ADNI’s demographic composition and scanner uniformity may limit the generalizability of our models to more heterogeneous clinical populations. To address this, future work will involve external validation on independent multi-center datasets such as AIBL [68], OASIS [69], and local hospital MRI cohorts to assess cross-population robustness. In addition, transfer learning and domain adaptation strategies will be explored to mitigate potential scanner- or site-specific biases, enabling broader deployment across diverse clinical environments.

We employed qualitative visualization-based interpretability to illustrate how the model focuses on clinically relevant brain regions. These brain-masked saliency maps are intended to provide intuitive insight into the spatial reasoning of the model rather than to serve as quantitative validation metrics. While quantitative saliency tests (e.g., sanity checks [70] or ROI-overlap analyses [71]) can further substantiate interpretability, such evaluations are beyond the present study’s scope.

## 6. Conclusions

This research demonstrates that lightweight deep learning models can achieve competitive performance in early Alzheimer’s disease detection while maintaining explainability and computational efficiency suitable for resource-constrained clinical settings. Our investigation yielded several key insights for the development of practical AI-based diagnostic tools. The comparison between MobileNetV2 and EfficientNetV2B0 revealed that modest increases in model complexity yield meaningful performance improvements. EfficientNetV2B0 with augmentation achieved 91.8% balanced accuracy compared to MobileNetV2 with augmentation at 90.0%, indicating that the efficiency–accuracy trade-off requires careful optimization rather than defaulting to minimal architectures. The effects of data augmentation varied a lot by architecture, with EfficientNetV2B0 benefiting substantially, while MobileNetV2 showed mixed results, highlighting the need for architecture-specific optimization strategies.

Our multi-scale attribution pipeline successfully validated that lightweight models learn clinically relevant features, focusing on hippocampal and ventricular regions consistent with established AD pathophysiology. The consensus mapping approach effectively combined coarse-grained regional importance with fine-grained textural features, providing interpretable explanations that align with clinical expectations and demonstrate that computational efficiency does not compromise anatomical relevance.

The 2D coronal slice methodology, while enabling deployment on standard clinical hardware, was necessitated by the computational demands of 3D volumetric analysis and inherently discards valuable 3D spatial relationships and inter-slice context. Additionally, our slice-level evaluation approach (600 test slices from 102 subjects; approximately 6 slices per subject) may overestimate model performance compared to subject-level aggregation due to potential correlation between slices from the same subject. While slice-level metrics provide robust statistical power for architecture comparison and are standard practice in medical imaging research with limited cohorts, clinical deployment scenarios would benefit from subject-level majority voting or probabilistic aggregation strategies to account for inter-slice dependencies and provide patient-level diagnostic confidence. Future work should implement subject-level evaluation protocols, quantify inter-slice correlation effects on performance estimates through intra-class correlation coefficient (ICC) analysis, and develop aggregation strategies that leverage full volumetric context while maintaining the computational efficiency demonstrated by our lightweight architectures. The limited dataset size of 102 subjects and homogeneous ADNI population constrains both statistical power and generalizability across diverse populations, scanner types, and clinical settings. Despite promising technical performance, significant gaps remain between research-grade models and clinically deployable tools, particularly with regard to real-world validation and integration with existing clinical workflows.

Based on these findings, several research directions address the identified limitations. Our immediate priority involves substantially expanding the dataset size and validation scope to include larger, more diverse multi-site datasets with heterogeneous populations and scanner protocols, establishing collaborations to assess performance across different acquisition standards and demographic groups where diagnostic disparities exist.

Developing computationally efficient 3D convolutional approaches that preserve spatial context while maintaining deployability constraints involves investigating progressive resolution training strategies and memory-efficient architectures to balance the benefits of volumetric analysis with resource limitations. Expanding our cross-sectional approach to longitudinal trajectory modeling involves developing frameworks capable of predicting conversion from stable mild cognitive impairment to progressive Alzheimer’s disease.

Systematic multimodal integration involves combining structural MRI with readily available clinical data such as cognitive assessments and demographic factors while preserving computational efficiency. Developing hierarchical fusion architectures that handle missing modalities in real-world scenarios strengthens the practical applicability. Establishing quantitative explainability frameworks beyond visual assessment involves collaborating with neuroimaging experts to develop metrics that measure attribution accuracy against known pathological patterns.

Developing a complete clinical decision support system integrated with existing radiology workflows is the ultimate goal. This requires extensive usability testing with clinicians, implementation of federated learning to address data-sharing constraints, and prospective clinical studies comparing AI-assisted workflows with standard diagnostic practices. These efforts aim to bridge the gap between laboratory results and practical tools that extend expert-level diagnostic capabilities to resource-constrained environments.

## Figures and Tables

**Table 1:** Comparison of diagnostic modalities for Alzheimer’s Disease.

Modality	Principle of Measurement	Key Findings in AD	Primary Advantages	Key Limitations
Structural MRI	Measures brain anatomy and volume using magnetic fields.	Atrophy in specific regions (hippocampus, temporal/parietal lobes).	Non-invasive, no radiation, widely available, high spatial resolution.	Traditionally measures late-stage neurodegeneration; less specific for underlying pathology.
Amyloid-PET	Visualizes extracellular Aβ plaques using a radiotracer.	Increased tracer uptake in neocortical regions.	High specificity for Aβ pathology; provides diagnostic clarity.	Expensive, limited availability, radiation exposure.
Tau-PET	Visualizes intracellular neurofibrillary tangles using a radiotracer.	Increased tracer uptake corresponding to Braak staging patterns.	High specificity for tau pathology; correlates well with cognitive decline.	Expensive, even more limited availability than amyloid-PET, radiation exposure.
FDG-PET	Measures regional brain glucose metabolism.	Hypometabolism in posterior cingulate/precuneus and temporoparietal lobes.	Sensitive indicator of neurodegeneration, often preceding structural atrophy.	Expensive, limited availability, radiation exposure, not specific to AD pathology.
CSF Analysis	Measures biomarker concentrations in cerebrospinal fluid via lumbar puncture.	Decreased Aβ42, increased t-tau and p-tau levels.	High diagnostic accuracy for Aβ and tau pathologies; direct measure of biochemistry.	Invasive procedure, risk of side effects, pre-analytical variability, lack of standardized cut-offs.
Cognitive Tests (MMSE/MoCA)	Brief, standardized tests of cognitive function (memory, attention, etc.).	Low scores indicating impairment in multiple cognitive domains.	Quick, inexpensive, non-invasive, accessible.	Low sensitivity for early/mild stages, influenced by education/culture, non-specific to cause.

**Table 2:** Summary of related works in Alzheimer’s disease classification using deep learning.

Study	Dataset	Model Architecture	Task	Explainability	Key Findings/Performance
**Deep Learning for AD Classification**					
Jo et al. (2019) [30]	ADNI	Various DL models	Binary (AD vs. CN)	Not Reported	Up to 98.8% accuracy on binary classification
Hechkel & Helali (2025) [34]	ADNI (sMRI + DTI)	YOLOv11	Multi-class detection	Not Reported	93.6% precision, 91.6% recall with multimodal data
Marcisz & Polanska (2023) [32]	ADNI	Various models	MCI vs. Early AD	Not Specified	Demonstrated sMRI-only feasibility
**Lightweight Architectures**					
Borah et al. (2024) [41]	Multiple medical imaging	MobileNet, VGG, ResNet	Multi-disease	Not Reported	Traditional models outperformed lightweight in some cases
Alruily et al. (2025) [42]	Not specified	Ensemble (VGG16, MobileNet, InceptionResNetV2)	AD classification	Not Reported	97.93% accuracy, 98.04% specificity via feature fusion
Cueto & Kelleher (2024) [40]	Various	Multiple architectures	Training efficiency	N/A	Efficiency vs. performance trade-off framework
**Explainable AI Methods**					
Huff et al. (2021) [44]	Review	Various CNNs	Medical imaging	Grad-CAM, saliency	Comprehensive XAI review
van de Leur et al. (2021) [47]	ECG data	Deep CNNs	ECG detection	Grad-CAM	Anatomical validation emphasis
Ennab & Mcheick (2025) [48]	Medical imaging	Various CNNs	Medical classification	Pixel-level, Grad-CAM	Comparative XAI analysis
Bhati et al. (2024) [49]	Survey	Various	Medical imaging	Multiple XAI	XAI visualization survey
**Multimodal Approaches**					
Kitamura & Topol (2023) [35]	Various	Multimodal AI	Medical imaging	Not focus	Multimodal AI in radiology
Schouten et al. (2024) [36]	Review	Multimodal	Medical AI	Not focus	Technical challenges review
**Current Study**					
**This Work**	**ADNI (102 subjects)**	**MobileNetV2, EfficientNetV2B0**	**Tri-class (CN, EMCI, LMCI)**	**Grad-CAM++, Guided Backprop**	**88% accuracy; lightweight interpretable early detection**

**Table 3:** Search criteria for ADNI MRI image selection.

Search Section	Criteria
Projects/Phase	ADNI, ADNI 1, ADNI GO, ADNI 2, ADNI 3, ADNI 4
Image Types	Pre-processed
Subject	Research Group - CN, EMCI, LMCI
Study/Visit	ADNI Baseline, ADNIGO Month 3 MRI, ADNI2 Baseline-New Pt, ADNI2 Month 6-New Pt, ADNI3 Initial Visit-Cont Pt
Image	Image Description - MPRAGE; Modality - MRI
Imaging Protocol	Acquisition Plane - 3D

**Table 4:** Complete preprocessing pipeline technical specifications for reproducibility.

Parameter	Specification
**Skull Stripping**	
Tool	SynthStrip v1.2 (FreeSurfer 7.4.1)
Success rate	100% (102/102 subjects)
**Spatial Preprocessing**	
Orientation	RAS canonical space (nibabel 5.1.0)
Native voxel spacing	1.0×1.0×1.2 mm^3^ (MPRAGE typical)
Resampling	None (native resolution preserved)
Template registration	None (subject-specific geometry preserved)
**Slice Extraction**	
Plane	Coronal ($i_{mid}\pm 15$)
Target per subject	30 slices
Actual yield	29.4 ± 0.8 slices/subject
Bounding box method	5th percentile intensity threshold
Morphological ops	Binary closing (disk radius = 5 pixels)
**Intensity Processing**	
Initial scaling	Native range → [0, 255] (8-bit)
MobileNetV2 normalization	(x/127.5)−1→[−1,1]
EfficientNetV2B0 normalization	x/255→[0,1]
DenseNet121 normalization	x/255→[0,1]
Application timing	On-the-fly during training
**Quality Control**	
Volume-level checks	Brain volume > 800 cm^3^, skull residual < 5%
Slice-level checks	Brain coverage > 30%, intensity 10–200
Total slices processed	3060 (102 subjects × 30 slices)
Slices excluded	60 (2.0%): 42 coverage, 18 artifacts
Subjects excluded	0 (0%)
Final dataset	3000 slices (1000 per class)

**Table 5:** On-the-fly data augmentation parameters applied during training.

Transformation	Range/Mode
Rotation	±10 degrees
Width Shift	±10% of image width
Height Shift	±10% of image height
Shear	±10%
Zoom	±10%
Horizontal Flip	Random
Brightness Adjustment	0.9–1.1
Fill Mode	Nearest

**Table 6:** Complete hyperparameter configuration for model training.

Parameter	Value
**Model Architecture**	
Input Image Size	224 × 224 × 3
Base Models	MobileNetV2, EfficientNetV2B0
Transfer Learning	ImageNet pre-trained weights
Custom Head	GAP + Dense (128, ReLU) + Dense (3, Softmax)
**Training Configuration**	
Batch Size	16
Learning Rate	$1\times 10^{-3}$
Optimizer	Adam ($\beta_{1}$ = 0.9, $\beta_{2}$ = 0.999)
Loss Function	Categorical Cross-Entropy
Early Stopping Patience	7 epochs
Maximum Epochs	100
**Hardware**	
GPU	NVIDIA A100
Training Time (MobileNetV2)	70–240 ms/step
Training Time (EfficientNetV2B0)	240–300 ms/step

**Table 7:** Inference performance metrics demonstrating clinical deployment readiness.

Model	Size (MB)	Params (M)	CPU (ms)	GPU (ms)	Studies/h
MobileNetV2 (Aug)	11.1	2.42	174.6	144.3	687
MobileNetV2 (Orig)	11.1	2.42	175.6	144.6	683
EfficientNetV2B0 (Aug)	25.4	6.08	345.5	314.1	347
EfficientNetV2B0 (Orig)	25.4	6.08	349.1	317.6	344
DenseNet121 (Aug)	29.8	7.17	505.6	452.0	237

**Table 8:** Per-class performance metrics for all model configurations.

Model	Class	Precision	Recall	F1-Score	Accuracy
EfficientNetV2B0 (Aug)	CN	0.93	0.83	0.88	0.8800
	EMCI	0.83	0.93	0.88	
	LMCI	0.89	0.88	0.88	
EfficientNetV2B0 (Orig)	CN	0.90	0.88	0.89	0.8750
	EMCI	0.85	0.90	0.87	
	LMCI	0.88	0.85	0.86	
MobileNetV2 (Aug)	CN	0.87	0.88	0.87	0.8650
	EMCI	0.85	0.86	0.86	
	LMCI	0.87	0.85	0.86	
MobileNetV2 (Orig)	CN	0.87	0.84	0.86	0.8600
	EMCI	0.84	0.88	0.86	
	LMCI	0.87	0.86	0.86	
DenseNet121 (Aug)	CN	0.88	0.78	0.82	0.8167
	EMCI	0.86	0.78	0.82	
	LMCI	0.74	0.90	0.81	

**Table 9:** Comprehensive discrimination and calibration metrics for all model configurations showing the impact of data augmentation on lightweight architectures.

Architecture	Configuration	Accuracy	Macro AUC [95% CI]	Micro AUC	Brier Score
EfficientNetV2B0	Original	0.8750	0.971 [0.961, 0.980]	0.971	0.0643
	**Augmented**	**0.8800**	**0.973 [0.963, 0.982]**	**0.973**	**0.0588**
MobileNetV2	Original	0.8600	0.962 [0.951, 0.972]	0.962	0.0706
	Augmented	0.8650	0.970 [0.961, 0.979]	0.970	0.0628
DenseNet121 ^†^	Augmented	0.8167	0.947 [0.933, 0.960]	0.942	0.0891

^†^ DenseNet121 was evaluated only in the augmented configuration as a deeper baseline (8 M parameters) for validating lightweight architecture performance. Bold indicates best overall performance. Lower Brier scores indicate better calibration.

**Table 10:** Class-wise sensitivity, specificity, positive predictive value (PPV), and negative predictive value (NPV) for all augmented model configurations.

Model	Class	Sensitivity	Specificity	PPV	NPV
EfficientNetV2B0 (Aug)	CN	0.835	0.970	0.933	0.922
	EMCI	0.930	0.905	0.830	0.963
	LMCI	0.875	0.945	0.888	0.938
	Macro Avg	0.880	0.940	0.884	0.941
MobileNetV2 (Aug)	CN	0.875	0.935	0.871	0.937
	EMCI	0.865	0.925	0.852	0.932
	LMCI	0.855	0.938	0.872	0.928
	Macro Avg	0.865	0.933	0.865	0.932
DenseNet121 (Aug)	CN	0.775	0.948	0.881	0.894
	EMCI	0.775	0.938	0.861	0.893
	LMCI	0.900	0.840	0.738	0.944
	Macro Avg	0.817	0.909	0.827	0.910

**Table 11:** Training convergence details for all model configurations.

Model Configuration	Convergence Epoch	Best Val. Accuracy	Final Test Accuracy
MobileNetV2 (Original)	27	0.8729	0.8600
MobileNetV2 (Augmented)	49	0.8708	0.8650
EfficientNetV2B0 (Original)	30	0.8521	0.8750
EfficientNetV2B0 (Augmented)	68	0.8792	0.8800
DenseNet121 (Augmented)	78	0.8354	0.8167

**Table 12:** Five-fold cross-validation performance summary showing mean accuracy and stability metrics.

Model Configuration	Mean Accuracy	Std Dev.	Min Accuracy	Max Accuracy
EfficientNetV2B0 (Augmented)	0.8800	0.0100	0.8650	0.8950
EfficientNetV2B0 (Original)	0.8750	0.0120	0.8580	0.8900
MobileNetV2 (Augmented)	0.8650	0.0110	0.8500	0.8800
MobileNetV2 (Original)	0.8600	0.0115	0.8450	0.8750
DenseNet121 (Augmented)	0.8100	0.0252	0.7800	0.8400

**Table 13:** Per-class performance metrics across 5-fold cross-validation (mean ± std).

Model	Class	Precision	Recall	F1-Score
EfficientNetV2B0 (Aug)	CN	0.93 ± 0.02	0.83 ± 0.03	0.88 ± 0.02
	EMCI	0.83 ± 0.04	0.93 ± 0.03	0.88 ± 0.02
	LMCI	0.89 ± 0.02	0.88 ± 0.04	0.88 ± 0.02
EfficientNetV2B0 (Orig)	CN	0.90 ± 0.02	0.88 ± 0.02	0.89 ± 0.01
	EMCI	0.85 ± 0.03	0.90 ± 0.03	0.87 ± 0.02
	LMCI	0.88 ± 0.02	0.85 ± 0.03	0.86 ± 0.01
MobileNetV2 (Aug)	CN	0.87 ± 0.02	0.88 ± 0.02	0.87 ± 0.01
	EMCI	0.85 ± 0.02	0.86 ± 0.03	0.86 ± 0.02
	LMCI	0.87 ± 0.02	0.85 ± 0.03	0.86 ± 0.01
MobileNetV2 (Orig)	CN	0.87 ± 0.02	0.84 ± 0.03	0.86 ± 0.02
	EMCI	0.84 ± 0.03	0.88 ± 0.02	0.86 ± 0.01
	LMCI	0.87 ± 0.02	0.86 ± 0.02	0.86 ± 0.01
DenseNet121 (Aug)	CN	0.83 ± 0.02	0.79 ± 0.03	0.81 ± 0.03
	EMCI	0.80 ± 0.04	0.80 ± 0.08	0.80 ± 0.05
	LMCI	0.80 ± 0.04	0.85 ± 0.04	0.82 ± 0.02

**Table 14:** Individual fold performance for EfficientNetV2B0 (augmented configuration) demonstrating cross-validation stability.

Fold	Accuracy	Balanced Accuracy	Convergence Epoch
1	0.8850	0.8845	62
2	0.8900	0.8897	75
3	0.8650	0.8651	58
4	0.8950	0.8943	71
5	0.8650	0.8655	74
**Mean ± Std**	0.88 ± 0.01	0.88 ± 0.01	68 ± 12

**Table 15:** Chi-square test results for pairwise model agreement analysis.

Model Pair	$\chi^{2}$ Statistic	*p*-Value	DoF	Agreement	Interpretation
MobileNetV2 vs. EfficientNetV2B0	537.66	<0.001	4	78.00%	Significant agreement
MobileNetV2 vs. DenseNet121	451.92	<0.001	4	73.83%	Significant agreement
EfficientNetV2B0 vs. DenseNet121	537.76	<0.001	4	77.33%	Significant agreement

**Table 16:** Contingency table for MobileNetV2 vs. EfficientNetV2B0 (augmented configurations).

EfficientNetV2B0∖MobileNetV2	CN	EMCI	LMCI	Total
**CN**	138	22	19	179
**EMCI**	26	173	25	224
**LMCI**	18	22	157	197
**Total**	182	217	201	600

Green shading indicates diagonal agreement where both models classified cases identically.

**Table 17:** Contingency table for MobileNetV2 vs. DenseNet121 (augmented configurations).

DenseNet121∖MobileNetV2	CN	EMCI	LMCI	Total
**CN**	130	26	20	176
**EMCI**	14	149	17	180
**LMCI**	38	42	164	244
**Total**	182	217	201	600

Green shading indicates diagonal agreement where both models classified cases identically.

**Table 18:** Contingency table for EfficientNetV2B0 vs. DenseNet121 (augmented configurations).

DenseNet121∖EfficientNetV2B0	CN	EMCI	LMCI	Total
**CN**	139	21	16	176
**EMCI**	16	154	10	180
**LMCI**	24	49	171	244
**Total**	179	224	197	600

Green shading indicates diagonal agreement where both models classified cases identically.

## Data Availability

Restrictions apply to the availability of these data. Data were obtained from the Alzheimer’s Disease Neuroimaging Initiative (ADNI) database and are available at https://adni.loni.usc.edu/ (accessed on 29 September 2025) with the permission of ADNI. This study did not generate any new data, and all analyses were performed on the existing ADNI dataset. The code used for the research can be found in https://github.com/falahsheikh/eAlz (accessed on 22 October 2025).
